# Allergy Modulation by N-3 Long Chain Polyunsaturated Fatty Acids and Fat Soluble Nutrients of the Mediterranean Diet

**DOI:** 10.3389/fphar.2020.01244

**Published:** 2020-08-21

**Authors:** Astrid Hogenkamp, Anna Ehlers, Johan Garssen, Linette E. M. Willemsen

**Affiliations:** ^1^Division of Pharmacology, Department of Pharmaceutical Sciences, Faculty of Science, Utrecht University, Utrecht, Netherlands; ^2^Center for Translational Immunology, University Medical Center Utrecht, Utrecht University, Utrecht, Netherlands; ^3^Department of Dermatology/Allergology, University Medical Center Utrecht, Utrecht University, Utrecht, Netherlands; ^4^Global Centre of Excellence Immunology, Danone Nutricia Research B.V., Utrecht, Netherlands

**Keywords:** n-3 long chain poly-unsaturated fatty acids, fat-soluble micronutrients, vitamins, polyphenols, allergy

## Abstract

The Mediterranean diet, containing valuable nutrients such as n-3 long chain poly-unsaturated fatty acids (LCPUFAs) and other fat-soluble micronutrients, is known for its health promoting and anti-inflammatory effects. Its valuable elements might help in the battle against the rising prevalence of non-communicable diseases (NCD), including the development of allergic diseases and other (chronic) inflammatory diseases. The fat fraction of the Mediterranean diet contains bioactive fatty acids but can also serve as a matrix to dissolve and increase the uptake of fat-soluble vitamins and phytochemicals, such as luteolin, quercetin, resveratrol and lycopene with known immunomodulatory and anti-inflammatory capacities. Especially n-3 LCPUFAs such as eicosapentaenoic acid (EPA) and docosahexaenoic acid (DHA) derived from marine oils can target specific receptors or signaling cascades, act as eicosanoid precursors and/or alter membrane fluidity and lipid raft formation, hereby exhibiting anti-inflammatory properties. Beyond n-3 LCPUFAs, fat-soluble vitamins A, D, E, and K1/2 have the potential to affect pro-inflammatory signaling cascades by interacting with receptors or activating/inhibiting signaling proteins or phosphorylation in immune cells (DCs, T-cells, mast cells) involved in allergic sensitization or the elicitation/effector phase of allergic reactions. Moreover, fat-soluble plant-derived phytochemicals can manipulate signaling cascades, mostly by interacting with other receptors or signaling proteins compared to those modified by fat-soluble vitamins, suggesting potential additive or synergistic actions by applying a combination of these nutrients which are all part of the regular Mediterranean diet. Research concerning the effects of phytochemicals such as polyphenols has been hampered due to their poor bio-availability. However, their solubility and uptake are improved by applying them within the dietary fat matrix. Alternatively, they can be prepared for targeted delivery by means of pharmaceutical approaches such as encapsulation within liposomes or even unique nanoparticles. This review illuminates the molecular mechanisms of action and possible immunomodulatory effects of n-3 LCPUFAs and fat-soluble micronutrients from the Mediterranean diet in allergic disease development and allergic inflammation. This will enable us to further appreciate how to make use of the beneficial effects of n-3 LCPUFAs, fat-soluble vitamins and a selection of phytochemicals as active biological components in allergy prevention and/or symptom reduction.

## Fatty Acids and Fat-Soluble Components of the Mediterranean Diet and Allergy Development

**Figure 1:**
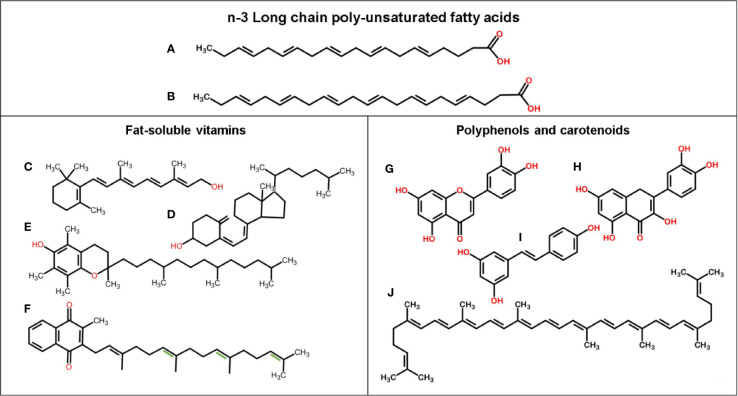
Chemical structure of n-3 LCPUFAs and fat-soluble bioactive components. **(A)** EPA, **(B)** DHA, **(C)** Vitamin A (retinol), **(D)** Vitamin D3 (cholecalciferol), **(E)** Vitamin E (alpha-tocopherol), **(F)** Vitamin K1 (phylloquinone), and with extra double bonds (in green) Vitamin K2 (menaquinone-4), **(G)** Luteolin, **(H)** Quercetin, **(I)** Resveratrol, and **(J)** Lycopene.

**Figure 2:**
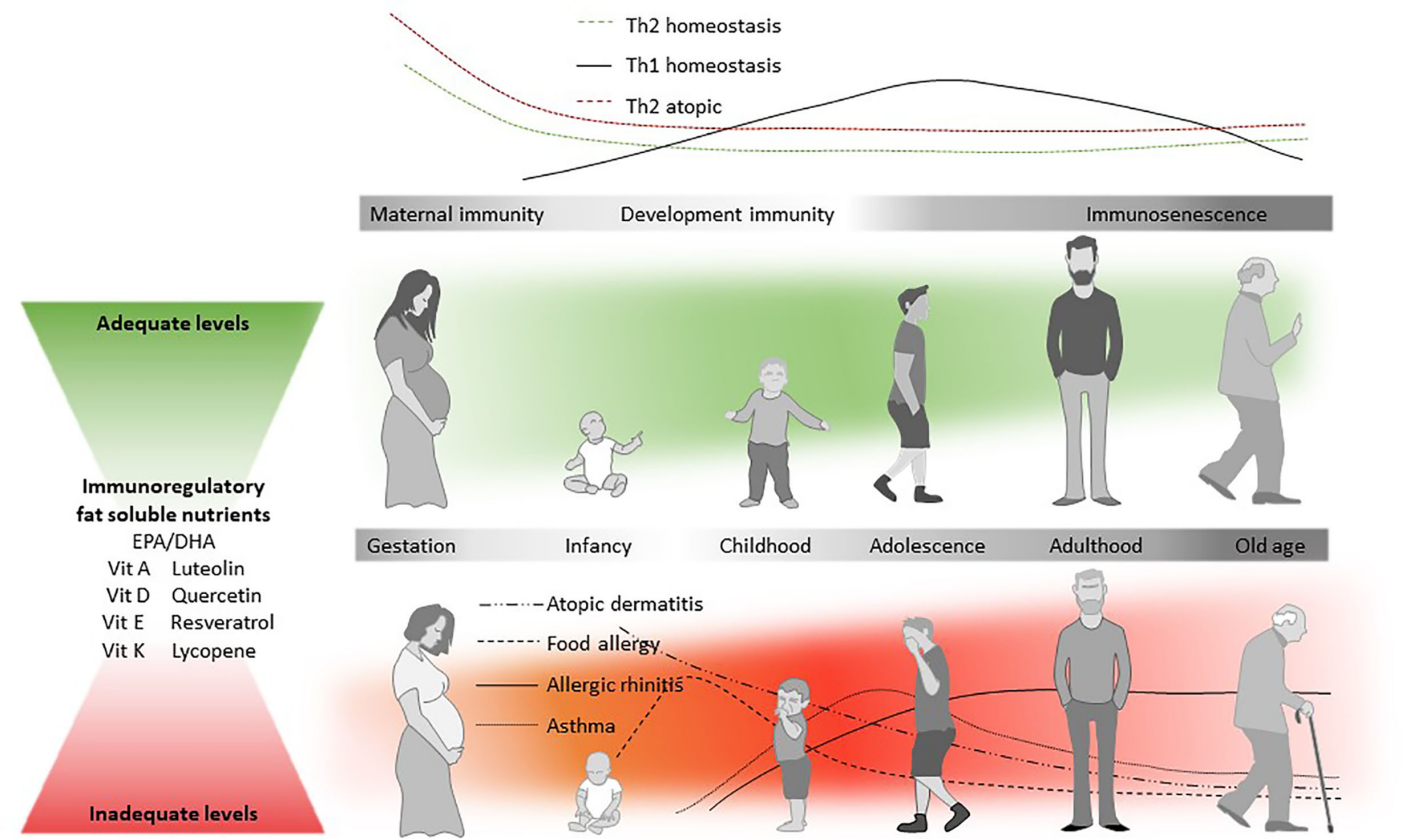
Diet and the atopic march. Immune responses are typically Th2-dominated in the early stages of life (top panel), but Th1-responses start developing after birth. In atopic individuals, Th2 responses tend to be higher already from an early age. The likelihood that an individual will develop allergic disease is partly determined by genetic predisposition, but genetic drift cannot explain the rise in prevalence (Agache et al., 2019). In the so-called Atopic March, atopic dermatitis (AD) is often the first clinical manifestation of allergic disease, which is typically followed by food allergy, rhinitis, and asthma (Spergel, 2010), all being characterized by a Th2 immune response. AD is followed by the development of allergen specific IgE (develop) and/or food allergy. In contrast to IgE-mediated food allergies, inhalant allergies and asthma are developed later in childhood. The increased susceptibility to atopic disease (red panel) is thought to be mediated at least in part by the nutritional environment during early development. Beyond other known immunoregulatory dietary components, n-3 LCPUFA and fat soluble nutrients may also help to reduce the allergy risk, and it is hypothesized that adequate levels (in green) of EPA/DHA, vitamin A, D, E, and K1/2, luteolin, quercetin, resveratrol, and lycopene could play an important role in maintaining immune-homeostasis throughout life.

Metabolic and immunological disturbances underlie the recent rise in non-communicable diseases (NCD). Beyond auto-immune diseases, the risk of allergic diseases and asthma has been steadily rising over the last decades, reaching the alarming prevalence of 5%–40% in Western populations (Agache et al., 2019). The Mediterranean diet is known for its health promoting and anti-inflammatory effects (Barbaresko et al., 2013; Sánchez-Sánchez et al., 2020). The fat component of the Mediterranean diet typically consists of olive and marine oils. Notably, particularly marine oil derived n-3 long chain poly-unsaturated fatty acids (LCPUFAs) such as eicosapentaenoic acid (EPA) and docosahexaenoic acid (DHA) may have immunomodulatory properties to mitigate unwanted inflammation and reduce the risk of allergy development (Venter et al., 2019). Additionally, the used oils may facilitate bioavailability of fat-soluble micronutrients such as vitamin A, D, K, E and phytochemicals such as polyphenols and carotenoids (**Figure 1**) (White et al., 2017). In contrast, n-9 mono-unsaturated oleic acid is olive oil’s main fatty acid, but it is not known for its anti-allergic properties. However, olive oils are also rich in anti-oxidants (e.g. tocopherols, carotenes), phenols, secoiridoids, lignans, and flavones (luteolin, apigenin) all known for their anti-inflammatory effects (Aludatt et al., 2017). Moreover, fruits and vegetables, accounting for a significant component of the Mediterranean diet, are rich sources of fat-soluble phytochemicals such as quercetin (flavonoid) in onions and apples, luteolin (flavonoid) in parsley, resveratrol (stilbene) in wine and berries and carotenoid lycopene in tomatoes (**Figure 1** and **Table 1**) (Mazzocchi et al., 2019). The uptake and bioavailability of these fat-soluble micronutrients may be enhanced in the presence of olive oil. They can be absorbed *via* inclusion in micelles required for fatty acid uptake by the intestinal epithelium and released basolaterally in chylomicrons which traffic *via* the lymphatics into the bloodstream (Boileau et al., 1999; Arranz et al., 2015; Mashurabad et al., 2017; White et al., 2017; Rinaldi de Alvarenga et al., 2019). Enhanced bioavailability of fat-soluble bioactive components may enhance health benefits, including protection against allergic inflammation. Indeed, allergy protective effects of the Mediterranean diet have been suggested in several observational studies, but thus far data have been inconclusive (Biagi et al., 2019). In early life, one of the first outcomes of allergic disease is atopic dermatitis and/or food allergy while later in childhood and during adolescence allergic rhinitis and asthma are more prevalent (**Figure 2**).

**Table 1:** Food sources for n-3 LCPUFAs and fat-soluble micronutrients.

**Nutrient**	**Food source**	**Reference**
**n-3 LCPUFAs**	Fatty fish such as salmon, mackerel, herring, and sardines Algae oil and fish oil	(Gebauer et al., 2006)
**Vitamin A**	As retinol: whole or semi-skimmed milk, eggs, and liver As carotenoids: carrots, spinach, chard, sweet potatoes (orange), pumpkins, mango, papaya	(Gilbert, 2013)
**Vitamin D3^1^**	Fatty fish such as salmon, mackerel, herring, and sardines Red meat, liver, and egg yolks	(Wolpowitz and Gilchrest, 2006)
**Vitamin E**	Plant oils such as olive, soya, and corn oil Nuts and seeds Wheatgerm such as in cereals	(Böhm, 2018)
**Vitamin K1**	Green leafy vegetables such as spinach, broccoli, parsley, lettuce, cauliflower, and cabbage Vegetable oils Cereal grains	(DiNicolantonio et al., 2015)
**Vitamin K2^2^**	Curd cheese, cheese, and fermented soy products (natto)	(Conly and Stein, 1992)
**Luteolin**	Parsley, celery, broccoli, and onions Cereals such as millet and wheat	(Lin et al., 2008)
**Quercetin**	Onions, curly kale, leeks, broccoli, and blueberries Red wine and tea	(Dabeek and Marra, 2019)
**Resveratrol**	Grapes, wine, peanut, and soy	(Burns et al., 2002)
**Lycopene**	Fruit such as guavas, watermelon, pink grapefruit, papaya Vegetables such as tomatoes, red peppers, asparagus, red cabbage	(Cruz Bojórquez et al., 2013)

^1^predominately produced in the skin from 7-dehydrocholesterol by UV light.

^2^produced by bacteria during fermentation and also by bacteria in the gut.

**Figure 3:**
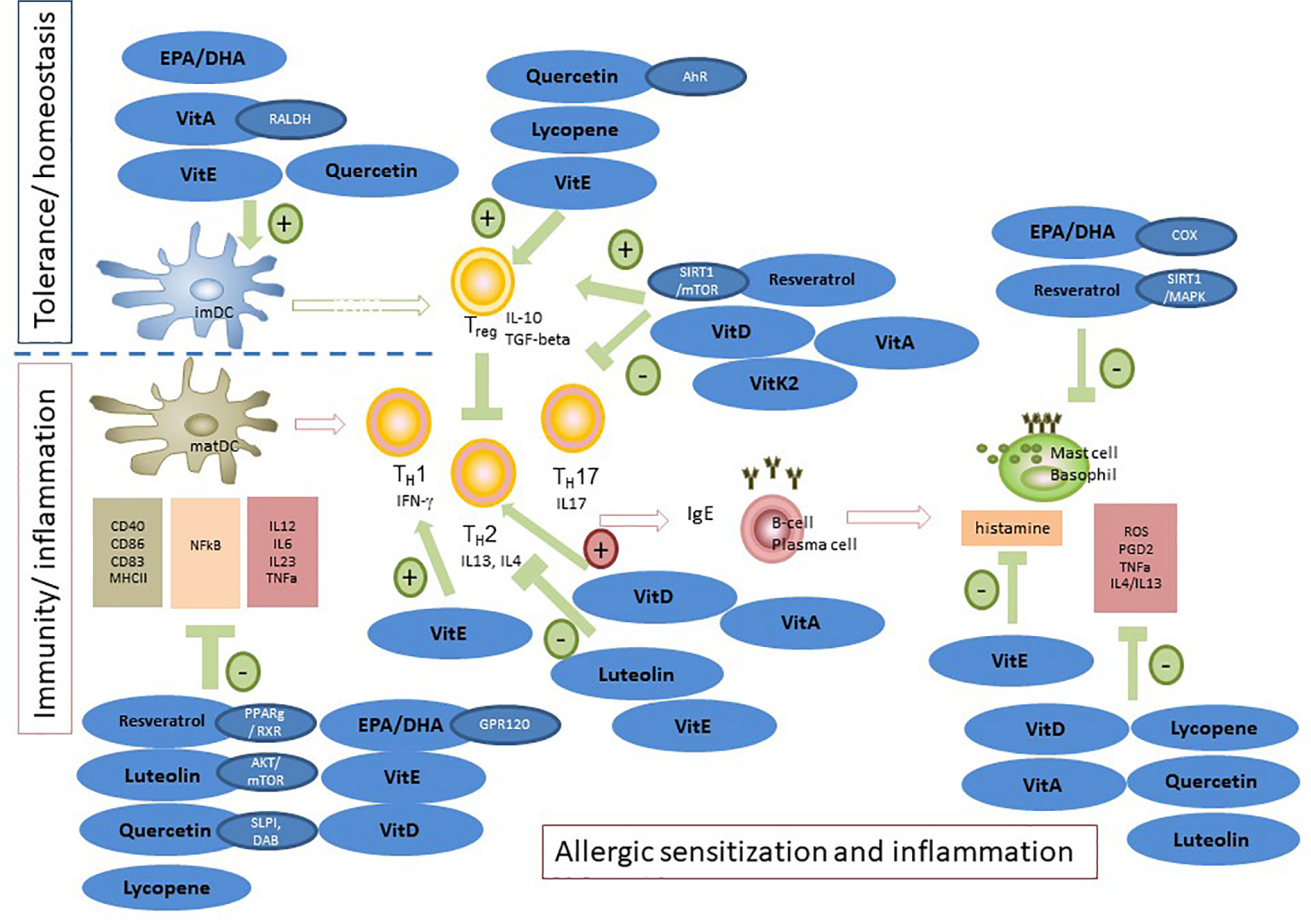
Modulation of allergic sensitization and effector phase by n-3 LCPUFAs and fat-soluble vitamins, polyphenols and carotenoids. In *in vitro* and pre-clinical studies, the potency of n-3 LCPUFAs and several fat-soluble micronutrients to instruct DC silencing was indicated, rendering DCs that support Treg development. In addition, LPS or inflammatory induced maturation of DCs can be suppressed by multiple of these nutrients, resulting in reduced proliferation and activation of consequent effector T-cells responses, hence attenuating pro-inflammatory responses. Also, Th2 driven allergy development can be mitigated by these micronutrients, either by directly suppressing Th2 development or *via* enhancing Treg or Th1 responsiveness, known to down regulate Th2 activation. In addition, mast cell or basophil activation is modified or suppressed in various ways by n-3 LCPUFA and the selected fat-soluble micronutrients. Some micronutrients play an ambivalent role since they can lower pro-inflammatory responses *via* enhancing not only Treg but also Th2 function (VitD and VitA). This may be a point of concern in case of allergic predisposition. Of note is that the beneficial immunomodulatory effects of vitamin E are mainly linked to the alpha-tocopherol form and even though not much is known about immune effects of VitK, the main immunomodulatory effects appear to relate to the VitK2 isoform. These micronutrients can act *via* several receptors or signaling transduction cascades and are frequently tested as single component for their immunomodulatory capacities. However, since they target similar cells involved in the allergic sensitization and effector cascade their effects may by additive or synergistic when combined aiming to prevent allergy development or reduce severity of symptoms.

Allergy evolves due to hampered immunological tolerance induction at mucosal sites such as the intestinal and pulmonary mucosa or the skin. Upon crossing this physical barrier, allergens are taken up by antigen-presenting cells such as dendritic cells (DCs) which subsequently present processed peptides originating from the allergens in a major histocompatibility complex class II (MHC-II) molecule to naïve T helper (Th) cells (Roche and Furuta, 2015). In a Th2 driving environment, characterized by increased interleukin (IL) 4 and IL-13 expression and/or release of epithelial factors such as IL-33, IL-25, and thymic stromal lymphopoietin (TSLP), these DCs differentiate into DC2s that instruct naïve Th-cells to differentiate into Th2-cells (Van Dyken et al., 2016). Interaction of these Th2-cells with naïve B-cells recognizing the allergen *via* the B-cell receptor and CD40-CD40 ligand co-stimulatory interaction supports the class-switch of naïve IgM+ B-cells to IgE+ B cells. Upon activation, these B-cells differentiate into IgE-secreting plasma cells (Iciek et al., 1997). These IgE-antibodies can be bound by the high-affinity FcϵRI receptor located on the surface of mast cells and basophils (effector cells) (**Figure 3**). Upon re-exposure, the allergen is recognized by IgE antibodies and cross-linking of at least two different FcϵRI receptors triggers the release of pre-formed (e.g. histamines) and *de-novo* synthesized mediators (e.g. lipid mediators like prostaglandins) and cytokines/chemokines driving allergic symptoms (Kambayashi and Koretzky, 2007).

## Modulation of Allergic Inflammation by N-3 LCPUFAs

**Figure 4:**
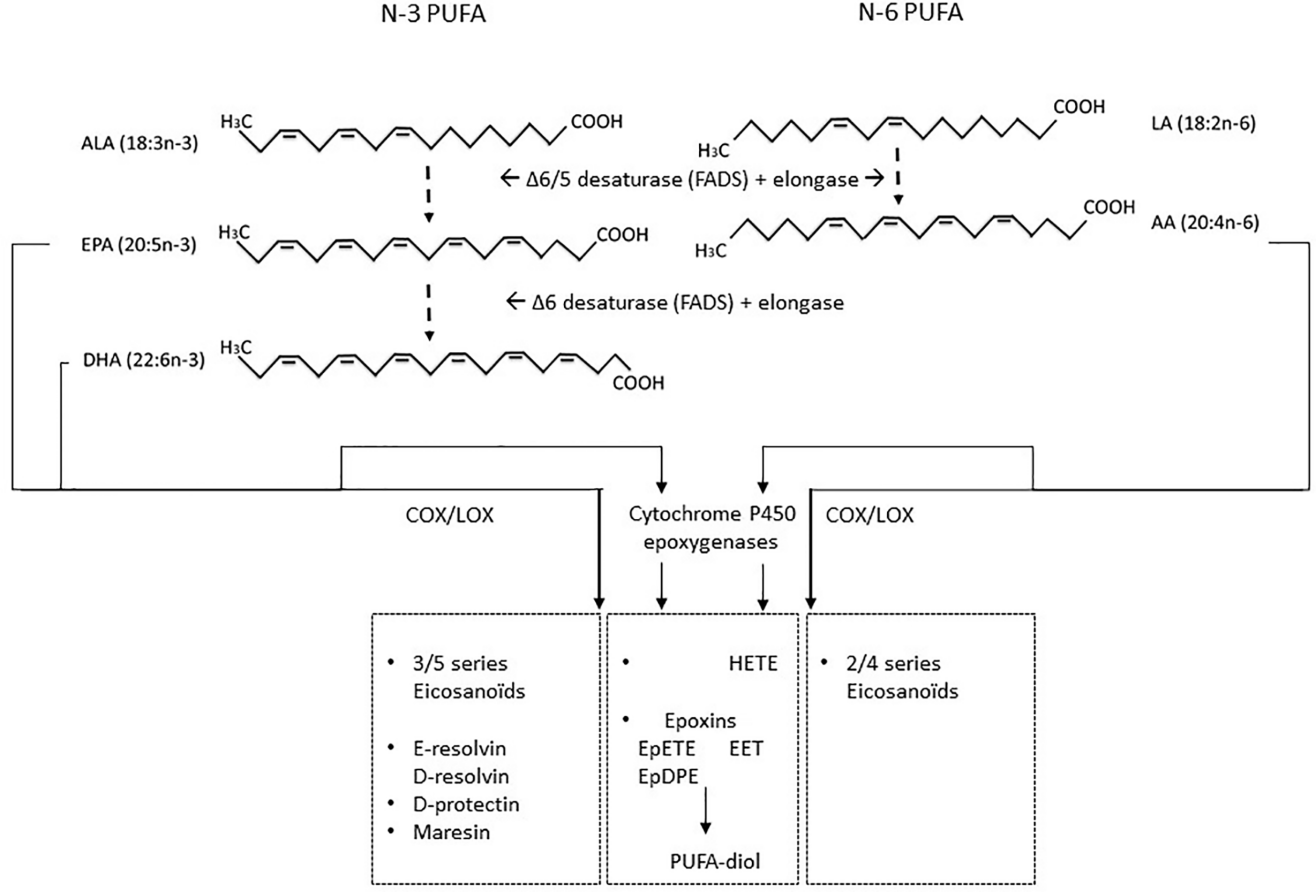
LCPUFAs and their metabolites N-3 ALA and n-6 LA are essential PUFAs that can be formed into either n-3 series of long chain PUFA EPA and DHA or n-6 LCPUFAs AA *via* fatty acid elongases and desaturases enzymes. These LCPUFAs are substrates for COX and LOX which can convert EPA into 3–5 series of eicosanoids or AA into 2–4 series of eicosanoids. In addition, EPA and DHA can be converted into anti-inflammatory protectins, resolvins, or maresins. Beyond COX and LOX, cytochrome P450 epoxygenases can convert n-6 AA or n-3 LA, EPA, and DHA in anti-inflammatory and cardiovascular protective epoxins such as epoxyeicosatrienoic acid (EET) derived from AA, and EPA or DHA derived epoxyeicosatetraenoic acid (EpETE) and epoxydocosapentaenoic acid (EpDPE). However, these may be further converted into PUFA-diols by soluble epoxidehydrolase, resulting in loss of their protective function and induction of possible harmful effects. In addition, cytochrome P450 epoxygenase conversion of AA can contribute to several classes of hydroxyeicosatetraenoic acid (HETE) which can promote inflammation.

Fatty fish are rich in n-3 LCPUFAs such as EPA (C20:5) and DHA (C22:6). EPA and DHA are essential components of cell lipid bilayers and play an important role in visual and neurodevelopment and cardioprotection (Miles and Calder, 2012; van den Elsen et al., 2012). In humans, these n-3 LCPUFAs can be synthesized from the essential fatty acid alpha-linolenic acid (ALA; C18:3) (present in vegetables, oils such as linseed oil and nuts such as walnuts) by fatty acid desaturase encoded by the *FADS* genes and elongase activity (**Figure 4**). However, the conversion rate of ALA into n-3 LCPUFA is only 10% or lower, depending on gene expression and activity of the rate limiting enzymes Δ5 or Δ6 desaturase and elongase (Tanaka et al., 2009). Additionally, the ratio of n-3 LCPUFAs to n-6 LCPUFA linoleic acid (LA, C18:2) and n-6 LCPUFA arachidonic acid (AA, C20:4) is unfavorably low (1:10–20) in the Western diet, due to its high content of n-6 PUFAs from vegetable oils such as sunflower and corn oil, eggs and meat [reviewed by (Miles and Calder, 2012; van den Elsen et al., 2012)]. A more preferable n-3:n-6 ratio would be 1:4 since n-3 ALA and n-6 LA compete for conversion by desaturases and elongase and also for inter-exchange in cell membranes phosphobilayers (Sprecher et al., 1995). Thus, a disbalance in favor of n-6 PUFAs may result in enhanced inflammatory responses since the n-6 LCPUFA AA will be converted by cyclo-oxygenase (COX) and lipoxygenase (LOX) enzymes into 2 and 4 series of prostaglandins (PGE2, PGD2) and leukotriens (LTB4), respectively (Barden et al., 2016). By contrast, n-3 LCPUFA EPA conversion by the same enzymes results in less inflammatory 3 and 5 series eicosanoids. Additionally, EPA and DHA are precursors for resolvins and maresins that restrict and silence inflammation, hence contributing to homeostasis [reviewed by (van den Elsen et al., 2012)]. LCPUFA can also be converted by cytochrome P450 epoxygenases, resulting in anti-inflammatory and cardioprotective epoxins such as epoxyeicosatrienoic acid (EET) derived from n-6 AA, and epoxyeicosatetraenoic acid (EpETE) and epoxydocosapentaenoic acid (EpDPE) from n-3 EPA or DHA respectively (Spector and Kim, 2016; Ostermann et al., 2019). In contrast, AA can also be converted in several pro-inflammatory hydroxyeicosatetraenoic acids (HETE) (reviewed by Calder, 2015). In addition, the epoxins may be further metabolized in PUFA-diols by soluble epoxidehydrolase which may cause undesirable side effects, related to—among others—increased oxidative stress responses (Fleming, 2019; dos Santos and Fleming, 2020). This underpins the importance of adequate levels of anti-oxidant intake when consuming high doses of LCPUFAs.

N-3 LCPUFAs EPA and DHA can also directly enhance anti-inflammatory signaling cascades [reviewed by (van den Elsen et al., 2012)]. DHA is a well-known ligand for the transmembrane G-protein coupled receptor 120 (GPR120) and intracellular receptor PPARγ. By binding to these receptors, DHA can generate its anti-inflammatory effect by lowering NFkB (nuclear factor kappa-light-chain-enhancer of activated B-cells) activation (Hara et al., 2011; Scirpo et al., 2015). Furthermore, n-3 LCPUFAs enhance membrane fluidity due to their high number of unsaturated bonds, thus impacting cellular function by altering lipid raft formation and cellular signaling (van den Elsen et al., 2012). However, the high amount of unsaturated bonds leads to the risk of oxidation and formation of reactive oxygen species (ROS). In this regard, fat-soluble vitamin E (tocopherol) has an important function, since it is present in the cell membrane and can scavenge formed radicals and prevent cell damage due to lipid peroxidation.

### N-3 LCPUFA and DCs and T-Cells

DCs are key players in host defense and the maintenance of tolerance (Steinman and Banchereau, 2007; Coquerelle and Moser, 2010). Several papers reported effects of EPA and DHA on DC functioning. In human monocyte-derived DCs (moDCs), DHA and EPA decreased expression of lipid presenting protein CD1, IL-6 secretion, and expression of GPR120 (Oh et al., 2010; Rajnavolgyi et al., 2014). Moreover, differentiation of human moDCs in the presence of EPA diminished lipopolysaccharide (LPS)-induced maturation and cytokine release (Zeyda et al., 2005). Correspondingly, moDCs treated with EPA have a decreased potency to stimulate allogeneic T-cells (Zeyda et al., 2005). These inhibitory effects were associated with a dose-dependent suppression of LPS-induced p38 mitogen-activated protein kinase (MAPK) phosphorylation by EPA and DHA (Wang et al., 2007). Furthermore, PPARγ target genes in human moDCs are induced by DHA-exposure, suggesting DHA to be involved in activation of the heterodimer of PPARγ and retinoid X receptor (RXR). PPARs are highly expressed in DCs, and DHA derivatives act as potent PPAR agonists (Yamamoto et al., 2005), resulting in lower IL-12 expression in murine bone marrow derived DCs (BMDCs). However, DHA-induced inhibition of NFκB p65 nuclear translocation appeared to be PPARγ-independent (Kong et al., 2010; Draper et al., 2011). Overall, treatment with DHA led to an immature DC phenotype (Kong et al., 2010). Interestingly, T-cells co-cultured with DHA-treated murine BMDCs expressed higher levels of transforming growth factor (TGF) β and forkhead box P3 (Foxp3), but a functional regulatory T-cell (Treg) phenotype was not found (Kong et al., 2010). In contrast, DHA treatment of murine DCs has been observed to increase expression levels of co-stimulatory molecules (Carlsson et al., 2015). However, T-cell stimulation was similarly inhibited, accompanied by an increased proportion of Treg in co-cultures with DHA-primed DCs (Carlsson et al., 2015). Hence, EPA and DHA have the capacity to affect the phenotype and function of DCs, which in turn can alter immune outcomes by lowering T-cell activation and inducing regulatory responses (**Figure 3**).

#### N-3 LCPUFA and B-Cells

In the presence of DHA, *ex-vivo* B-cell IgE class-switching (induced by anti-CD40 and IL-4) is reduced in peripheral blood mononuclear cells (PBMCs) from AD patients (Koch et al., 2008). Potentially, IgE class-switching ability is reduced by direct interference with CD40 and IL-4 signaling pathways, subsequently specifically blocking transcription of the epsilon germline transcript (ϵGLT) in DHA-treated PBMCs from non-atopic patients (Weise et al., 2011). Moreover, *ex-vivo* treatment of PBMCs from non-atopic donors with DHA metabolites reduced transcription of ϵGLT by stabilizing B-cell lymphoma 6 protein (Bcl-6), acting as a suppressor of the ϵGLT transcription factor STAT6. However, due to low numbers of IgE-producing B-cells, significant suppression of class-switching was not confirmed (Kim et al., 2015).

#### N-3 LCPUFA and Mast Cells

**Figure 5:**
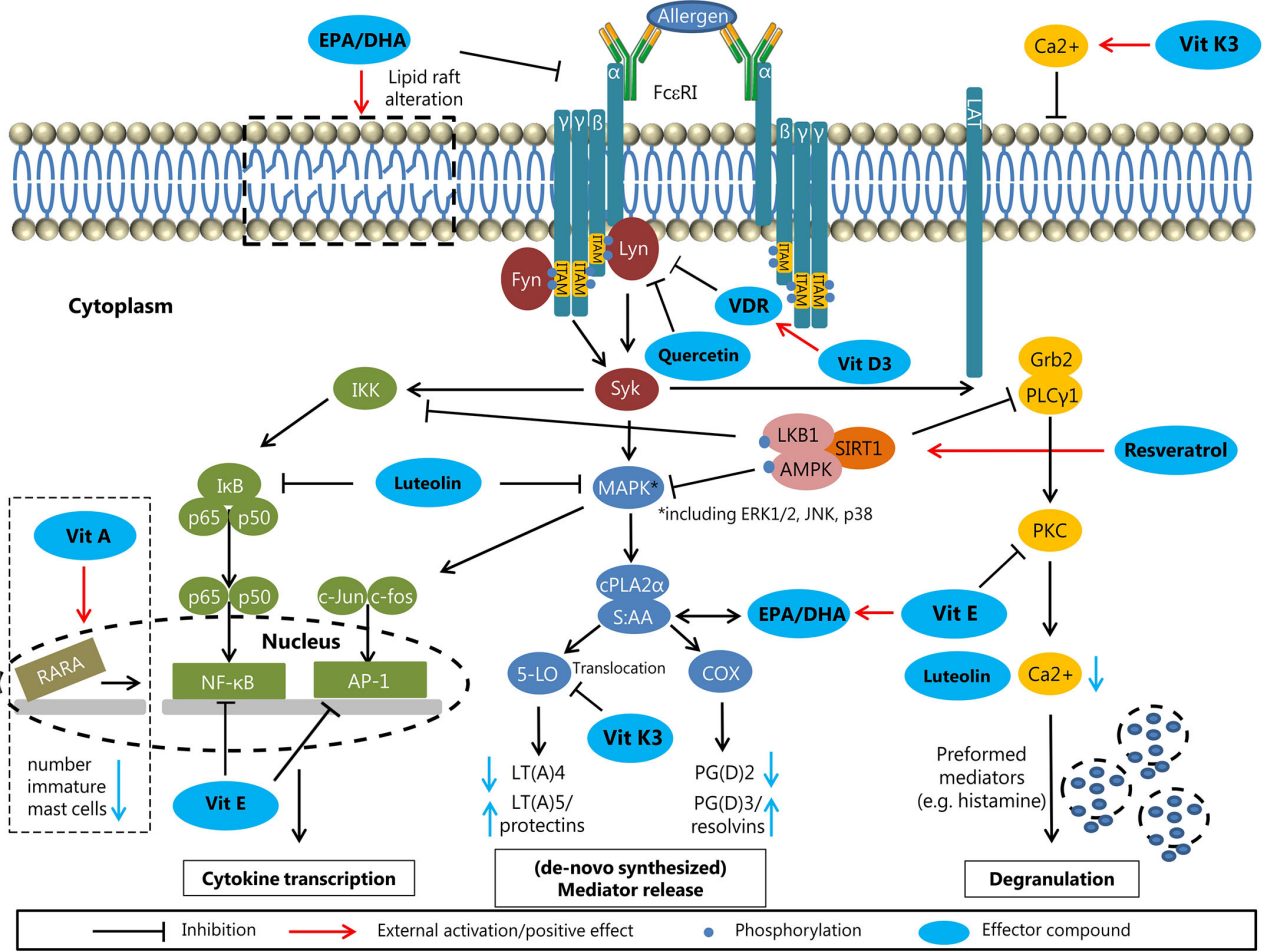
Molecular effects of n-3 LCPUFAs and micronutrients on effector cells (effector compounds are highlighted in blue). *N-3 LCPUFAs*: Alteration of the lipid raft composition as indicated at the top left, potentially affecting the assembly of FcϵRI signaling proteins and attenuating the FcϵRI signaling. Fatty acids from the phospholipid bilayer are used for the formation of lipid mediators. Replacing AA by EPA or DHA results in the production of less inflammatory mediators (LT(A)5/protectins and PG(D)2/resolvins). *Vitamins:* The fat-soluble vitamins A, D, and E can influence mast cell signaling at different stages. Vitamin A influences the gene transcription by RARA in the nucleus, resulting in lower numbers of immature mast cells. The metabolite vitamin D3 from vitamin D can upregulate its receptor VDR, resulting in inhibition of Lyn, an upstream protein of the FcϵRI signaling cascade. Vitamin E has the potential to inhibit the transcription factors NFκB and AP-1, resulting in less (pro-inflammatory) cytokine transcription. *Phytochemicals:* The flavonoids luteolin and quercetin have directly inhibitory effects on FcϵRI signaling by affecting Lyn (Quercetin) and MAPK and IκB (luteolin) upstream of the cytokine transcription and mediator forming. The stilbene resveratrol can activate the inhibitory Sirt1 complex, resulting in less cytokine transcription, less mediator forming and reduced degranulation potency. Synthetic vitamin K3 (not allowed in human) is converted into vitamin K2 in the intestine.

Cross-linking of high affinity FcϵRI receptors on effector cell membrane leads to the release of (lipid) mediators. Pre-incubation with n-3 LCPUFAs EPA and/or DHA dose-dependently reduced PGD2 production by human cord blood derived mast cells (CBMCs) and HMC-1 cells compared to AA pre-incubation by competing for conversion *via* COX (Obata et al., 1999; van den Elsen et al., 2013a). This reducing effect was also observed in NC/Nga mice (PGE2 and LTB4) (Suzuki et al., 2002; Yoshida et al., 2016). Moreover, n-3 LCPUFAs reduce production of pro-inflammatory cytokines such as IL-4 and IL-13, probably by decreasing ROS levels and further downstream inhibition of MAPK phosphorylation without affecting NFκB (van den Elsen et al., 2013a). In contrast, ROS production was increased in a canine mastocytoma cell line (C2) after pre-administration of AA and EPA. Adding antioxidants like vitamin E (VitE) diminished this activating effect of EPA (Schmutzler et al., 2010). Administration of n-3 LCPUFAs may also decrease Th2 cytokine expression by inhibiting transcription factor GATA-1 phosphorylation in murine mast cells (Park et al., 2013). However, *in-vivo* experiments on n-3 LCPUFAs supplementation in allergy management are controversial which might be explained by experiments performed by Shimanaka et al. (Matsuda et al., 1997; Sierra et al., 2008; Jang et al., 2018; Lee et al., 2019). Phospholipase PAF-AH2 knock-out mice (C57BL and BALB/c) with an impaired conversion of n-3 LCPUFAs into n-3 epoxins showed attenuated allergic symptoms compared to wild-type mice upon n-3 LCPUFAs supplementation and antigen-induced activation was restored by applying n-3 epoxins. Double knockout of PAF-AH2 and Src-kinase-signaling inhibitor 1 (Srcin 1) in bone marrow-derived mast cells (BMMCs) also restored antigen-induced activation, suggesting an important role of Srcin 1 in negatively regulating FcϵRI signaling, being inhibited by n-3 epoxins (Shimanaka et al., 2017). N-3 LCPUFAs also modulate lipid raft compositions in C57BL/6 BMMCs, resulting in more fluid membrane regions and altered assembling of signaling proteins. Fat-1 mice, converting n-6 to n-3 PUFAs (43), show an abnormal shuttling of FcϵRI within lipid rafts at baseline compared to C57BL/6 mice (Wang et al., 2015). Overall, n-3 LCPUFAs may negatively modulate mast cell activation by interfering at different stages, although the tendency to be oxidized, potentially leading to mast cell activation, should be considered and may be circumvented by supplementing antioxidants (**Figure 5**).

#### N-3 LCPUFAs in Pre-Clinical Allergy Models

Allergic sensitization itself has been shown to decrease serum triacylglycerol levels of EPA and DHA in mice (Ruhl et al., 2008), which may be linked to altered lipoprotein distribution (Schweigert, 2001). House dust mite (HDM) sensitized asthmatic mice had increased serum levels of AA, while di-homo-γ-linolenic acid was decreased (Fussbroich et al., 2019). In a study by Sierra *et al*, contact dermatitis was induced in Balb/c mice after a 3-week dietary intervention with diets containing fish oil (Sierra et al., 2008), with either 1.42% EPA or 1.42% DHA of total fatty acid content compared to the control diet. Although, both diets reduced ear inflammation (Sierra et al., 2008), the EPA-rich diet reduced the local and systemic inflammatory response and Th2 responses, while increasing IL-10 production (Sierra et al., 2008). In contrast, a diet supplemented with 5% EPA was not able to prevent AD-like dermatitis in NC/Nga mice (Lee et al., 2019) developing AD-like symptoms spontaneously (Matsuda et al., 1997). These contrasting outcomes may be explained by differences in experimental models and mouse strains, although Fat-1 mice were recently observed to exhibit markedly reduced AD-like skin lesions (Jang et al., 2018) suggesting that EPA and DHA play a protective role in AD. Cow’s milk, hen’s egg and peanut are major food allergens (Sicherer et al., 2010). In mice with orally induced hen’s egg allergy [ovalbumin (OVA)], a 13% salmon oil diet (6.1% EPA and 7.5% DHA) partially prevented development of food allergic symptoms (Hogenkamp et al., 2011) although no differences in immunoglobulin levels or effector cell-populations were observed. However, a 7% fish oil diet (15.9% EPA and 7.9% DHA) provided directly after weaning of mice prevented increase in OVA-specific IgE and IgG1 levels after subcutaneous sensitization and oral challenges with OVA (de Matos et al., 2012). Furthermore, edema, eosinophil infiltration and mucus production in the proximal jejunum were reduced in the fish oil group (de Matos et al., 2012). In mice with orally induced cow’s milk allergy (CMA), allergic sensitization and whey-induced symptoms were largely prevented by a diet in which 6% of the soybean oil was replaced by tuna oil (7.0% EPA and 27.8% DHA) (van den Elsen et al., 2013c). Mice fed the tuna oil diet had lower serum whey specific IgE levels, and Th2 and Th1-cell frequency in the mesenteric lymph nodes (MLN) and/or spleen, in sham and whey sensitized mice. Also increased frequencies of tolerogenic DCs in MLN and Foxp3+Tregs in spleen and intestinal lamina propria were observed in sensitized tuna oil fed mice (van den Elsen et al., 2013c). Adoptive transfer of these splenic Tregs protected naïve mice from allergy development (van den Elsen et al., 2013b; van den Elsen et al., 2013c). In a similar follow-up study, EPA (28.8% EPA and 13.7% DHA) or DHA (7% EPA and 27.8% DHA) rich fish oil were compared in whey or peanut sensitized mice (van den Elsen et al., 2014). Both fish oil diets attenuated the acute allergic skin response to whey, but peanut specific skin responses were only suppressed in mice fed the DHA enriched diet. Oral peanut sensitization was not prevented by either of the oils, but whey-specific IgE and IgG1 levels were lower in mice fed the DHA-enriched diet (van den Elsen et al., 2014). Hence, the quality of the fish oil (i.e. the ratio of the n-3 LCPUFAs) is of importance for its potential to prevent food allergy (van den Elsen et al., 2014). Asthma is a chronic inflammatory disorder of the respiratory tract, characterized by reversible airflow obstruction, bronchial hyperresponsiveness, and eosinophil and T-cell infiltration in the lungs (Lemanske and Busse, 2010). In murine OVA-induced allergic pulmonary inflammation, menhaden fish oil supplementation reduced pulmonary oxidative stress. Remarkably, fish oil supplementation enhanced the production of IL-5, IL-13, and reduced production of protective pulmonary PGE2 (Yin et al., 2009). Oral fish oil supplementation in a rat model for allergic airway inflammation lowered concentrations of nitrite lipid hydroperoxide, and increased activities of superoxide dismutase and glutathione peroxidase (Zanatta et al., 2014). However, fish oil did not affect proinflammatory platelet activating factor (PAF) bioactivity in lung tissue, suggesting a dual effect of fish oil on oxidative stress and inflammation in asthma (Zanatta et al., 2014). In contrast, OVA-induced allergic airway inflammation led to less severe airway inflammation and bronchoconstriction in Fat-1 mice (Bilal et al., 2011). Higher levels of endogenous n-3 LCPUFAs, and the counter-regulatory mediators protectin D1 (PD1) and resolvin E1 (RvE1) were measured in Fat-1 mice lung tissue. PD1 and RvE1 have anti-inflammatory and pro-resolving effects (Serhan et al., 2008). Interestingly, another DHA-derived pro-resolving mediator maresin-1 also suppressed murine allergic airway inflammation (Krishnamoorthy et al., 2015). Maresin-1 augmented *de-novo* generation of Tregs interacting with type 2 innate lymphoid cells (ILC2s) to suppress cytokine production in a TGF-β–dependent manner (Krishnamoorthy et al., 2015). In line with earlier observations (Bilal et al., 2011), OVA-specific Th-cell activation and proliferation was suppressed as a consequence of reduced T-cell receptor signaling in Fat-1 mice (Jang et al., 2014). Additionally, lower Th1 and Th2 cytokine levels in bronchoalveolar lavage fluid (BALF) of OVA-sensitized and challenged mice were found (Jang et al., 2014). Dietary fish oil intervention (6.3% menhaden oil containing 13% EPA, 12% DHA, and 0.7% soybean oil) prevented OVA-induced airway inflammation, hyperresponsiveness, eosinophilia, and airway fibrosis, accompanied by a reduction of OVA-specific IgE and Th2 and Th17 type cytokines in the lung (Bargut et al., 2013).

#### N-3 LCPUFAs in Allergy Prevention in Human Observational Studies

N-3 LCPUFAs blood levels in early life have been linked to allergy protection in several studies. In infants between 6 and 14 months of age, cord blood EPA and DHA and total n-3 LCPUFAs correlated negatively with AD development (50% risk reduction) (Montes et al., 2013). Breastmilk DHA levels correlated with asthma prevention at 4 years of age (60% risk reduction), and the risk for AD was reduced by 50% at 1 year and 4 years of age. However, this effect was only observed in atopic mothers with n-3 LCPUFAs levels above the median (Wijga et al., 2006). Moreover, high breastmilk n-3 LCPUFAs levels, protected against sensitization for inhalant allergens in children up to 2 years of age (50% risk reduction) (Rosenlund et al., 2016). High content of long chain fatty acids and double bond numbers in maternal blood triacylglycerols was associated with up to 40% risk reduction of food allergy in the offspring (Hong et al., 2019). High n-3 LCPUFAs plasma levels at 3 years of age (and n-6 LCPUFAs), were inversely associated with asthma and/or wheeze and allergic sensitization for inhalant and food allergens, which was most pronounced in children with high cord blood 25-hydroxyvitamin D at birth (≥ 50% risk reduction) (Lee-Sarwar et al., 2019). Imprinting effects of early n-3 LCPUFAs exposure were still observed in children at 6–7 years of age (Stratakis et al., 2018). Strikingly, n-3 LCPUFAs EPA and DHA, and a higher total n-3/n-6 LCPUFA ratio were associated with a risk reduction in wheeze and asthma of approximately 50%, but not with allergic rhinitis or AD. However, in another recent study, higher n-3 LCPUFAs blood levels at 8 years of age were associated with a 20-30% risk reduction for aeroallergen sensitization, rhinitis and asthma (Magnusson et al., 2015). Of note, n-6 LCPUFA AA was also linked with allergic asthma and rhinitis protection and remission of airway allergies within this period. In young adults, n-3 LCPUFAs plasma levels were no longer associated with protection against airborne and food allergens sensitization (Woods et al., 2004). Interestingly, asthma severity was negatively associated with total n-3 LCPUFAs plasma levels and bronchial hyperreactivity with DHA, while n-6 LCPUFAs did not show protective effects. Also in adults diagnosed with asthma, EPA blood levels were associated with a lower risk of non-specific bronchial hyperresponsiveness (aOR 0.66), while AA increased this risk (aOR 1.21) (Adams et al., 2018). In addition, in serum or PBMCs of adult AD patients, n-6 LCPUFA patterns were found to differ from healthy controls, and in the skin AA levels and some of its metabolites were significantly increased (Lindskov and Holmer, 1992; Mihály et al., 2013; Mihály et al., 2014; Töröcsik et al., 2019).

These observational studies consistently show strong positive associations between cord blood or plasma n-3 LCPUFAs levels in current and long-term allergy protection during infancy. In addition, altered LCPUFA status and enhanced AA levels are linked with current asthma or AD in adults. However, although these studies do correct for lifestyle factors, no direct causal relationships can be drawn from observational studies.

#### N-3 LCPUFAs in Placebo Controlled Dietary Intervention Studies

Placebo-controlled dietary intervention studies mostly use fish oil as an n-3 LCPUFAs source, and early life intervention already starts during early gestation. In the DOMinO trial, pregnant women (n=706) with a familiar risk of allergy were provided daily with 900 mg n-3 LCPUFAs (1.5 g fish oil DHA concentrate) starting from 21 weeks of gestation until delivery, but this intervention did not protect the offspring against airborne and food allergen sensitization, nor allergy (rhinitis, eczema, wheeze) at 3 or 6 years of age (Best et al., 2018). However, at 1 year of age, hen’s egg sensitization was reduced by more than 30%, and at 1–3 years of age the prevalence of AD was lowered by 25% (tendency p=0.10) (Palmer et al., 2012; Palmer et al., 2013). In an earlier study, atopic women (n=98) were provided a higher dose n-3 LCPUFAs (3.7 g) starting from week 20 of gestation until delivery. This resulted in a mean increase of 4% n-3 LCPUFAs in the erythrocyte membranes of the neonates at the expense of n-6 LCPUFA AA, and slightly reduced *ex-vivo* allergen specific cytokine release of cord blood mononuclear cells (CBMNCs) (Dunstan et al., 2003b). Additionally, hen’s egg sensitization (skin prick test) was reduced by more than 50% and although the frequency of AD at 1 year of age was unaltered, children in the fish oil group had significantly less severe symptoms with the Scoring atopic dermatitis (SCORAD) test. IL-13 plasma levels were also lower in the neonates, correlating with an increase of n-3 LCPUFAs incorporated in cell membranes (Dunstan et al., 2003a). Daily administration of 0.65g n-3 LCPUFAs (fish oil) during pregnancy enhanced mRNA levels of regulatory TGF-β in both maternal blood and cord blood, while IL-1 and interferon (IFN)-γ plasma levels were reduced in the mothers and IL-4, IL-13 were lowered in the neonatal cord blood (Krauss-Etschmann et al., 2008). The protective effect of n-3 LCPUFAs may result from epigenetic changes when provided early in life or during pregnancy, hereby affecting the health outcome also later in life. Daily administration of 400 mg DHA starting from day 18–22 of gestation until delivery resulted in enhanced methylation of both IL-13 and IFN-γ gene transcription sites compared to the control group, hence lowering their expression (Lee et al., 2013). The preventive effect of n-3 LCPUFA intervention may further increase when also provided during breastfeeding. Daily maternal supplementation with 2.7 g n-3 LCPUFAs starting from week 25^th^ of gestation until 3^rd^–4^th^ month of breastfeeding in an at risk population significantly lowered the prevalence of AD and food allergy in the offspring at 1 year of age (Furuhjelm et al., 2009). Even though this encouraging study concerned a small cohort, it appeared that the dosing, timing, and duration of maternal intervention may have contributed to this successful prevention of allergy in some of these children. This protective effect was still measured at 2 years of age, although new cases were not prevented. Children (or their mothers) with the highest EPA and DHA plasma levels had the lowest chance of developing allergy and severity of developed allergies was reduced (Furuhjelm et al., 2011). Providing asthmatic women or their offspring with a lower daily dose of n-3 LCPUFAs (158 mg from week 36 of gestation until 6 months of breastfeeding or formula feeding) did not prevent allergic sensitization. However, it reduced wheeze and bronchodilator use especially in offspring with relatively high n-3 LCPUFAs plasma levels at 18 month and atopic cough at 3 years of age (Mihrshahi et al., 2004; Peat et al., 2004). In another study, high risk children were supplemented with n-3 LCPUFAs (390 mg) from birth until 6 months of age. Although HDM IgE levels were reduced at 1 year of age, the children were not protected against food allergy or wheeze. However, infants without wheeze at 1 year of age had significantly higher n-3 LCPUFAs plasma levels and lower allergen specific Th2 responses (D’Vaz et al., 2012a; D’Vaz et al., 2012b). Formula milk with added DHA and n-6 LCPUFA AA reduced the risk of all allergies in children from the general population, while the prevalence of wheezing was reduced in children at risk in the first year of life (Foiles et al., 2016). Furthermore, daily supplementation of 2.4 g n-3 LCPUFAs (fish oil) in 736 pregnant women from week 24 of gestation until 1 week after delivery reduced the infant’s risk on pediatrician diagnosed persistent wheeze or asthma for 30% at 3 to 7 years of age. This was associated with reduced risk of lower respiratory tract infections as compared to a control group supplemented with olive oil (Bisgaard et al., 2016). The protective effect was particularly evident upon supplementation of women with low EPA and DHA blood levels (<4.3%), resulting in a risk reduction of more than 50%. Low blood levels were due to low n-3 LCPUFAs intake and a specific *FADS* genotype hampering LCPUFA conversion. The n-3 LCPUFAs supplementation appeared to be less effective in children receiving high dose of vitamin D3 (Bisgaard et al., 2016). In asthmatic infants (8–12 years), daily n-3 LCPUFAs (1.2 g) supplementation for 6 months reduced inflammatory marker TNF-α in plasma. However, no clinical benefit was observed although blood eosinophilia tended to reduce (p=0.011) (Hodge et al., 1998). Similar results were obtained in adult allergic asthmatics [5 weeks daily 0.7 g n-3 PUFA (630 mg DHA+EPA)], although pro-inflammatory markers as exhaled nitric oxide and the number of sputum and serum eosinophils reduced (Schubert et al., 2009). Pollen-induced hay fever and asthma in adults was also not prevented by a 6 month intervention with daily 5.4 g of EPA and DHA (Thien et al., 1993). These studies indicate that early life intervention with n-3 LCPUFAs may help to reduce the asthma risk, but treatment of allergic asthma using n-3 LCPUFAs is not effective in lowering symptoms. In contrast, daily supplementation of 5.2 g of EPA and DHA for 3 weeks did ameliorate exercise-induced bronchoconstriction in asthmatics, as determined by improved lung function and reduced bronchodilator use. This was associated with reduced sputum concentrations of pro-inflammatory LTC4-LTE4 and LTB4, PGD2, IL-1β and TNF-α and an increase on LTB5 (Mickleborough et al., 2006). A more recent study showed a protective effect when using 3.1 g per day (Williams et al., 2017).

Hence, n-3 LCPUFA may modulate allergic sensitization and/or inflammation as indicated in these clinical trials. Fat-soluble components like vitamins, polyphenols, stilbenes, and carotenoids may be capable of adding to these effects, since pre-clinical studies clearly show their potential in allergy protection.

## Effects on Allergic Inflammation by Fat-Soluble Micronutrients

### Vitamin A

Vitamin A (VitA) plays a key role in various processes such as embryogenesis (Metzler and Sandell, 2016) and immune function (Julia et al., 2015). This dietary component is obtained from plants as carotenoids (β-carotene) or from animal-derived food sources as retinyl esters or all-trans-retinol (Blomhoff and Blomhoff, 2006; Li et al., 2014). After uptake, VitA is eventually oxidized to all-trans-retinoic acid (RA) by retinal dehydrogenases (RALDHs) (Blomhoff and Blomhoff, 2006) expressed in epithelial cells (Lampen et al., 2000; Iwata et al., 2004) and DCs (Iwata et al., 2004; Coombes et al., 2007) in the gut. RA is a high-affinity ligand for the nuclear receptors RA receptor-α (RARα), RARβ, and RARγ forming heterodimers with RXR. RXR/RAR heterodimers can bind to RAR response elements (RAREs) or RXR response elements (RXREs) in the promoter regions of target genes, thereby controlling gene transcription in various cells including immune cells (Chambon, 1996; Raverdeau and Mills, 2014). In addition, carotenoids can affect the NFκB pathway, which decreases DNA-binding activity and inhibits inflammatory cytokines (Kaulmann and Bohn, 2014).

#### Vitamin A and DCs and T-cells

VitA is important for DC and T-cell functioning. Intestinal DCs express high levels of RALDH2, allowing them to synthesize RA (Iwata et al., 2004; Coombes et al., 2007). RA regulates the development and homeostasis of intestinal CD11b+(CD103+)+CCR7+ DCs (Klebanoff et al., 2013), which can induce gut-tropism and gut-homing in T-cells by inducing CCR9 (C-C chemokine receptor 9) and α4β7 expression (Johansson-Lindbom et al., 2003; Mora et al., 2003; Iwata et al., 2004). Importantly, DC-derived RA enhanced TGF-β driven differentiation of naïve T-cells into Tregs (Coombes et al., 2007; Mucida et al., 2007) and inhibited development of Th17-cells (Xiao et al., 2008). Moreover, VitA inhibits Th1 and supports Th2 differentiation (Iwata et al., 2003) by inducing IL-4 expression (Lovett-Racke and Racke, 2002), but it may also indirectly promote Th2 differentiation by modulating DCs (Hoag et al., 2002). Favoring Th2 responses in the gut can stimulate protective effects on the gut mucosa, but it may also enhance allergic responses (Schuster et al., 2008; Matheu et al., 2009). However, VitA deficiency has been shown to increase airway hyperreactivity (AHR) in humans (Chen et al., 2014) and MLN DCs from vitamin-A deficient mice induce inflammatory Th2-cells (Yokota-Nakatsuma et al., 2014). Furthermore, RA supplementation inhibited detrimental Th17 responses, while promoting Treg responses in a murine asthma model (Zhao et al., 2013). It is possible that these contrasting outcomes depend on the RA availability, i.e. RA may induce Tregs and inhibit or promote Th17 differentiation dependent on the concentration of biologically available RA (Julia et al., 2015) (**Figure 3**).

#### Vitamin A and Mast Cells

Compared to other vitamins, VitA surprisingly has a potential pro-inflammatory effect on mast cells. *Ex-vivo* stimulation of human-derived skin mast cells with RA increased the secretion of IL-1β, IL-6, IL-8, and TNF-α in a dose-dependent manner by binding of RA to the RARα (Babina et al., 2015). One of the most important effects of this mechanism is proliferation restriction in mast cells whereby immature mast cells are the dominated target (Kinoshita et al., 2000; Hjertson et al., 2003). The anti-proliferative trait of RA has been described for human mast cells *in* and *ex-vivo*, HMC-1-cells and murine peritoneal mast cells (Astorquiza et al., 1980; Alexandrakis et al., 2003; Ishida et al., 2003). Moreover, RA can enhance degranulation of human-skin derived mast cells, probably due to the wide range of genes regulated by RARα (Babina et al., 2017). Overall, the potential effect of VitA in humans must be critically evaluated since VitA can in fact diminish the number of mast cells, but has a potential pro-inflammatory effect. The combination with VitD and VitE might have beneficial impact on mast cell stabilization since these vitamins can negatively regulate FcϵRI signaling and subsequently diminish degranulation (**Figure 5**).

#### Vitamin A in Pre-Clinical Allergy Models

As outlined above, VitA and its metabolite RA can affect immune responses substantially and may therefore have impact on allergic outcomes. In a murine model for OVA-induced allergic asthma, RA supplementation attenuated airway inflammation and decreased Th17 and Th2 differentiation and functions while promoting Treg differentiation, which is partly in contrast to *in-vitro* observations (Wu et al., 2013). Lower Th17 and greater Treg responses were also observed by Zhao et al. in a chronic asthma model (Zhao et al., 2013). Interestingly, RA supported OVA-specific oral tolerance induction in OVA-sensitized mice and reduced OVA-induced AHR of recipient mice transferred with pulmonary Th-cells of tolerized RA treated donor mice, implying an immune imprinting effect of RA (Sakamoto et al., 2015). In line with these findings, VitA deficiency exacerbated lung inflammation and type 2 cytokine production in a similar murine model for allergic asthma (Cui et al., 2016). However, others have reported that disease severity in allergic asthma can be attenuated by VitA deficiency, while excessive VitA intake exacerbates pulmonary hyperresponsiveness (Schuster et al., 2008), indicating a small therapeutic window for VitA.

#### Vitamin A in Human Allergy Prevention

The impact of VitA supplementation on human allergic diseases has recently been reviewed by Hufnagl et al. (Hufnagl and Jensen-Jarolim, 2019). Lower levels of serum VitA have been observed in asthmatic children and adults (Allen et al., 2009; Nurmatov et al., 2011), although other studies do not confirm this (Hamalainen et al., 2017). Maternal intake of VitA has been suggested to lower the risk of asthma in children (Maslova et al., 2014), although other studies did not show this (Checkley et al., 2011; Nwaru et al., 2011). Neonatal supplementation with VitA is recommended in areas with VitA deficiency (serum (plasma) retinol <0.35 µmol/l), but this does not affect the risk of atopy (Kiraly et al., 2013). Similarly, dietary VitA supplementation in children does not affect allergic outcomes (Kim et al., 2016), although a systematic review showed weak but nonetheless supportive effects of VitA in asthma prevention (Nurmatov et al., 2011). In studies involving adults, VitA supplementation was provided in the context of dietary interventions such as the Mediterranean diet, but not as a single component (Hufnagl and Jensen-Jarolim, 2019). Increasing the dietary intake of fruit and vegetables rich in carotenoids can improve lung function and reduce risk of asthma exacerbation, associated with decreased systemic inflammation (Wood et al., 2012).

### Vitamin D3

Humans acquire vitamin D (VitD) primarily from exposure to sunlight. Vitamin D3 (VitD3) is synthesized in the skin from a derivative of cholesterol (Holick, 2007). In addition, VitD3 (or VitD2) can be obtained from the diet. In the liver, 25-hydroxylase (CYP2R1) hydroxylates VitD3 to 25-dihydroxyvitamin D3 (25(OH)D3), which is measured to assess VitD status (DeLuca, 2004). 25(OH)D3 is metabolically inactive and must be hydroxylated in the kidneys by 1-a-hydroxylase (CYP27B1) to 1,25(OH)2D3 (Holick et al., 1972). Interestingly, CYP27B1 is expressed in various immune cells, but only macrophages and some DCs also express CYP2R1, allowing these latter cell types to convert VitD3 to 1,25(OH)2D3 (Monkawa et al., 2000; Fritsche et al., 2003; Chen et al., 2007; Sigmundsdottir et al., 2007).

1,25(OH)2D3 binds to the Vitamin D receptor (VDR), a member of the nuclear receptor superfamily (Kutner and Brown, 2018). VDR heterodimerizes with nuclear receptors of the RXR family after binding to 1,25(OH)2D3, allowing the VDR-RXR heterodimer to bind Vitamin D Responsive Elements (VDREs) (Kutner and Brown, 2018). Depending on the target gene, this induces or represses gene transcription (Baeke et al., 2010; Haussler et al., 2011).

#### Vitamin D3 and DCs and T-Cells

1,25(OH)2D3 decreases expression of MHC class II molecules and of CD40, CD80 and CD86 on DCs, thereby downregulating differentiation, maturation and immunostimulatory capacity (Penna and Adorini, 2000; Griffin et al., 2001; Fritsche et al., 2003; van Etten and Mathieu, 2005). 1,25(OH)2D3 produced by DCs also leads to skin tropism by inducing CCR10 expression in T-cells (Sigmundsdottir et al., 2007), suggesting DCs can respond to local metabolites and subsequently direct the imprinting of tissue-specific tropism in T-cells (Iwata et al., 2004; Dudda et al., 2005; Sigmundsdottir et al., 2007). Additionally, 1,25(OH)2D3 suppresses IL-12 (D’Ambrosio et al., 1998; Penna and Adorini, 2000) and increases IL-10 production by DCs (Penna et al., 2006), suggesting that 1,25(OH)2D3 can steer DC-driven differentiation of T-cells, but 1,25(OH)2D3 also suppresses IL-2 expression in T-cells, potentially inhibiting their proliferation (Lemire et al., 1984; Lemire et al., 1985; Alroy et al., 1995). Furthermore, VitD3 treatment results in polarizing murine T-cells towards a Th2 phenotype (IL-4 release) (Boonstra et al., 2001), but others did not confirm this (Staeva-Vieira and Freedman, 2002). Furthermore, 1,25(OH)2D3 can suppress IL-6 and IL-23 synthesis (Penna et al., 2006; Daniel et al., 2008), which decreases Th17-responses. Interestingly, 1,25(OH)2D3 can also induce FoxP3 expression in T-cells (Penna et al., 2005; Gorman et al., 2007; Daniel et al., 2008), which—taken together with the findings discussed above—suggests that 1,25(OH)2D3 induces regulatory responses while inhibiting Th1 and Th17 immune outcomes. However, it is not clear whether involvement of 1,25(OH)2D3-driven DC responses are necessary for Treg induction as this is also observed in absence of DCs (Barrat et al., 2002; Jeffery et al., 2009) (**Figure 3**).

#### Vitamin D3 and Mast Cells

In epidemiological studies, deficiency of VitD has been associated with severe asthma, and spontaneous mast cell release is increased in VitD-deficient BALB/c mice (Poon et al., 2013; Liu et al., 2017). Vice versa, BALB/c mice fed a VitD-supplemented diet exhibited lower levels of histamine and TNF-α, indicators of mast cell release, compared to mice fed VitD-deficient or VitD-sufficient diets, suggesting stabilization of mast cells by VitD (Liu et al., 2017). This stabilization might be explained by upregulation of VDR expression by 1,25(OH)_2_D *in-vitro* (Babina et al., 2000; Baroni et al., 2007; Yip et al., 2014) VDR, in turn, binds to the tyrosine-protein kinase Lyn resulting in decreased phosphorylation of Syk and activation of the MAPK complex and NFκB in murine BMMCs. Moreover, VitD decreased TNF-α levels by binding to the promotor region of the TNF-α transcript (Liu et al., 2017). Impaired TNF-α expression upon VitD supplementation has also been observed in human CBMCs (Yip et al., 2014). These effects on mast cell modulation suggest a potential treatment option to attenuate allergic symptoms, especially in patients with severe reactions (**Figure 5**).

#### Vitamin D3 in Pre-Clinical Allergy Models

VitD deficiency affects lung function and volume in mice, which may affect pulmonary health (Zosky et al., 2011). Indeed, in mice sensitized to OVA at 8 weeks of age, perinatal VitD deficiency increased the capacity of airway-draining lymph node cells to proliferate in response to OVA stimulation *ex-vivo* (Gorman et al., 2013). Although increases in OVA-specific cytokine production were observed, pulmonary cell infiltration, BALF cytokine levels and serum IgE levels were unaffected by VitD deficiency (Gorman et al., 2013). In contrast, VitD deficiency worsened AHR, pulmonary eosinophilia, increased BALF pro-inflammatory cytokines and reduced IL-10 levels and lowered numbers of Tregs in OVA-sensitized mice at 6 weeks of age (Agrawal et al., 2013). Furthermore, VitD supplementation attenuated the pro-inflammatory effects, although allergic airway inflammation was not completely reversed (Agrawal et al., 2013). The contrast between these two studies may be explained by the timing and doses for OVA sensitization. Perinatal VitD deficiency induces Th2 skewing and a reduction of IL-10 secreting Tregs (Vasiliou et al., 2014). This was further enhanced in HDM-sensitized mice at one week of age. Although VitD insufficiency in early life did not affect AHR in this study, eosinophilic inflammation and airway remodeling was more severe in VitD-deficient mice sensitized to HDM. VitD supplementation after weaning reduced serum IgE levels, pulmonary eosinophilia and airway remodeling (Vasiliou et al., 2014). OVA-specific IgE and IgG1 levels were increased in VitD deficient mice (Heine et al., 2014), and co-administration of 1,25(OH)2D3 enhanced OVA-specific immunotherapy indicated by reduced allergic airway inflammation and AHR. However, treating OVA-sensitized mice with intraperitoneal 1,25(OH)2D3 at the time of intranasal OVA-challenge reduced allergic inflammation in non-deficient mice (Lai et al., 2013; Wang et al., 2016). Hence, indicating an important role in maintaining pulmonary homeostasis and allergic asthma protection.

VitD deficiency also exacerbated food allergic symptoms in mice sensitized intraperitoneally to OVA, which was suggested to be mediated by increased expression of IL-4 in MLN (Matsui et al., 2018). VitD deficiency could be involved in the development of food allergy, potentially by modulating immune responses and maintaining intestinal microbe homeostasis (Vassallo and Camargo, 2010). In humans, full VitD3 deficiency is unlikely, however reduced serum VitD3 levels are often observed.

#### Vitamin D3 in Human Allergy Studies

VitD status has been linked to differences in geographical locations with different degrees of sun exposures (Yeum et al., 2016). Interestingly, sun exposure has been inversely related to food sensitization during infancy (Matsui et al., 2015), suggesting a link between VitD status and risk of allergic sensitization. However, data from studies are inconclusive. Some studies report that allergic sensitization is more common in children and adolescents with low 1,25(OH)2D3 levels (Sharief et al., 2011; Baek et al., 2014). Furthermore, low cord blood VitD levels have been associated with increased cow’s milk sensitization but not with asthma, AD, or allergic rhinitis in early childhood (Chiu et al., 2014). Additionally, VitD insufficiency increased the risk of developing food allergies up to 11-fold in infants of Australian parents, depending on the allergen (Allen et al., 2013). Others have made similar associations as higher rates of VitD deficiency were found in children with persistent egg allergy (Neeland et al., 2018). In contrast, high maternal 1,25(OH)2D3 status has also been associated with increased risk of allergic disease in the offspring (Hansen et al., 2015). Another study reported that VitD deficiency during the first 6 months of infancy was not associated to an increased risk for food allergy at 1 year of age (Molloy et al., 2017). Moreover, no convincing associations between prenatal VitD status and allergic outcomes in childhood were found in a recent meta-analysis (Pacheco-Gonzalez et al., 2018).

The effects of (maternal) supplementation with VitD on food allergy development are also conflicting. One study reported inverse associations between maternal intake of VitD and sensitization to food allergens at 5 years of age (Nwaru et al., 2010). In contrast, in a randomized, double-blind, placebo-controlled trial maternal supplementation with VitD did not improve infant AD but rather appeared to increase the risk of developing food allergy (Norizoe et al., 2014). To further complicate matters, VitD supplementation during pregnancy increased the risk of food allergy in the offspring, whereas food-derived VitD during pregnancy was associated with a decreased risk (Tuokkola et al., 2016). However, no correlation between maternal VitD supplementation during pregnancy and food sensitization in the offspring at 2 years of age were found (Savage et al., 2018).

VitD-related outcomes for pulmonary (allergic) diseases appear to differ from food allergic outcomes. In a long-term prospective study, early life 1,25(OH)2D3 deficiency was associated with increased risk of persistent asthma at 10 years of age (Hollams et al., 2017; Pfeffer and Hawrylowicz, 2018), possibly resulting from the association between 1,25(OH)2D3 deficiency and increased risk for early allergic sensitization and upper respiratory tract colonization with bacterial pathogens (Hollams et al., 2017). Based on a systematic review and meta-analysis, VitD supplementation reduces the rate of asthma exacerbations in patients requiring treatment with systemic corticosteroids (Jolliffe et al., 2017). Furthermore, maternal VitD supplementation reduced the risk of persistent wheeze in the offspring throughout the first 3 years of life (Chawes et al., 2016). Additionally, VitD tended to reduce the incidence of asthma and recurrent wheezing in children from pregnant women at risk (Litonjua et al., 2016). In conclusion, it appears that—depending on the window of opportunity and the overall VitD status—VitD would contribute to maintaining homeostasis and may counteract the development of allergic disease.

### Vitamin E

VitE is the umbrella term for four tocopherols (α–δ- tocopherol) and four tocotrienols (α–δ-tocotrienol) (Traber, 2007), mainly available in edible oils (Slover, 1971). α-Tocopherol and γ-tocopherol are most abundant in food, and although the intake of γ-tocopherol from the diet is generally higher, α-tocopherol is predominantly found in mammalian plasma and tissues (Wolf, 2006). Both α-tocopherol and γ-tocopherol are lipid peroxyl radical scavengers, making them potent antioxidants (Jiang, 2014). Additionally, γ-tocopherol is capable of detoxifying nitrogen dioxide and peroxy-nitrite (Jiang, 2014). However, these tocopherols also affect signal transduction (e.g. modifying protein C kinase (PKC) activity (Mahoney and Azzi, 1988)). The outcome is dependent on the isoform, as α-tocopherol acts as an antagonist and γ-tocopherol is an agonist of PKC, leading to opposing roles in inflammation (Cook-Mills, 2013). α-Tocopherol can stimulate cyclic adenosine monophosphate (cAMP) production in human PBMCs, thereby attenuating proinflammatory cytokine and chemokine production (Salinthone et al., 2013). Moreover, VitE can affect the activity of many transcription factors like PPARγ and NFκB *via* modulation of signal transduction enzymes (Zingg, 2015). Interestingly, lipid rafts can also be altered by VitE, resulting in altered membrane protein interaction and translocation, modified signal transduction (Zingg, 2015). Overall, VitE appears to modulate cell functioning at multiple levels which cannot only be explained by its antioxidant function.

#### Vitamin E and DCs and T-Cells

VitE reduces human moDCs activation upon proinflammatory cytokine stimulation (Tan et al., 2005) and the capacity of DCs to induce T-cell proliferation, resulting in generation of anergic T-cells that have regulatory properties, suggesting a role for VitE in tolerance induction (Tan et al., 2005). Similarly, α-tocopherol exposure of murine BMDC lowered LPS-induced maturation (Xuan et al., 2016). Interestingly, maternal supplementation with α-tocopherol to allergic female mice reduced numbers of pulmonary CD11b+ DCs, but not CD11b− DCs (Abdala-Valencia et al., 2014), while γ-tocopherol exerts an opposing effect (Abdala-Valencia et al., 2016). This points to an important role of the different isoforms of VitE, as shifts in different subsets of DCs could affect maintenance of tolerance and the development of allergy. Notably, high average human plasma γ-tocopherol levels (relatively abundant in soy bean oil) are reported in countries with the highest asthma prevalence (Abdala-Valencia et al., 2014). The effects of VitE on T-cells have been studied in relation to immunosenescence, where VitE showed improved proliferative ability of old T-cells (Lee and Han, 2018). Furthermore, re-stimulated human PBMCs were protected from apoptosis in the presence of VitE accompanied by a reduction in CD95L expression, suggesting a protection from activation-induced cellular death (Li-Weber et al., 2002). VitE also dose-dependently reduced IL-4 production in activated human peripheral T cells (Li-Weber et al., 2002). Interestingly, this effect was observed both in T-cells isolated from allergic patients as well as in T-cells from non-allergic donors (Li-Weber et al., 2002), suggesting a role for VitE in modulating the Th1/Th2 balance (Han et al., 2006) (**Figure 3**).

#### Vitamin E and Mast Cells

In an allergic dermatitis mouse model (NC/Nga), scratching behavior, epidermis thickness and serum histamine levels upon sensitization were lower in mice fed a VitE supplemented diet (Tsuduki et al., 2013). Additionally, OVA-sensitized Brown Norway rats experienced less severe reaction upon challenge after treatment with VitE between sensitization and challenge, accompanied by reduced eosinophil infiltration (Wagner et al., 2008). As a potential mechanism, *in-vitro* experiments (HMC-1) showed inhibition of NFκB predominately reducing phosphorylation of protein kinase B (PKB). Hence, VitE can directly interfere with FcϵRI signaling (Kempna et al., 2004) (**Figure 5**).

#### Vitamin E and Pre-Clinical Allergy Mouse Models

Use of experimental animal models has led to further insight in the role of VitE on allergy. In a model for OVA-induced allergic airway inflammation, pulmonary levels of IL-5 and plasma levels of IgE were blunted in VitE deficient allergic mice (Lim et al., 2008). However, no differences in pulmonary eosinophils were observed, suggesting that the effects of VitE deficiency are most pronounced for early sensitization to allergens (Lim et al., 2008). The observed effects are possibly induced by Th1 skewing, as VitE supplementation has been shown to enhance antiviral Th1 responses in old mice (Han et al., 2000). In addition, a dose-dependent reduction of allergic inflammation in the offspring of α-tocopherol supplemented allergic mice was observed (Abdala-Valencia et al., 2014), whereas lung eosinophilia, inflammatory mediators and inflammatory CD11b+ DCs increased in offspring of γ-tocopherol supplemented allergic mice (Abdala-Valencia et al., 2016). In addition, daily administration of α-tocopherol during the OVA-challenge decreased pulmonary eosinophils and monocytes infiltration, γ-tocopherol treatment however increased airway inflammation (Berdnikovs et al., 2009). Similar anti-inflammatory effects of α-tocopherol supplementation have been described linking to protection against mitochondrial dysfunctions related to asthmatic inflammation (Mabalirajan et al., 2009). Interestingly, in mice treated with both γ-tocopherol and α-tocopherol, the beneficial effects of α-tocopherol were inhibited (Berdnikovs et al., 2009). These proinflammatory effects of γ-tocopherol could be partly reversed by supplemental levels of α-tocopherol (McCary et al., 2011).

#### Vitamin E in Human Allergy Studies

In a recent systematic review, maternal supplementation with VitE during pregnancy was found to reduce odds of asthma development and was negatively associated with childhood wheezing (Wu et al., 2018). However, the opposing effects of α-tocopherol and γ-tocopherol were not considered in this study. In asthmatics, α-tocopherol levels in airway fluid were reduced, although plasma concentrations were normal (Kelly et al., 1999; Kalayci et al., 2000). Supplementation with α-tocopherol of asthmatic patients decreased allergic inflammation and AHR (Hoskins et al., 2012), although older studies demonstrated mixed effects on wheeze (Troisi et al., 1995; Dow et al., 1996; Weiss, 1997; Smit et al., 1999; Tabak et al., 1999). These differential outcomes may reflect the opposing effects of α-tocopherol and γ-tocopherol, as beneficial effects were found in Italy and Finland (low average γ-tocopherol levels), but not in the United States or Netherlands (high average γ-tocopherol levels) (Cook-Mills et al., 2013). Overall, α-tocopherol has anti-inflammatory effects with potential clinical significance for the treatment of allergic lung diseases, but dietary patterns should be considered.

### Vitamin K

Vitamin K (VitK) exists in two natural forms, phylloquinone (K1) and menaquinone (K2), and in a synthetic form named menadione (vitamin K3). The number of studies examining the effects of VitK on immune functioning is limited, therefore, this section will briefly discuss VitK in relation to allergy (development).

In human PBMCs, VitK2, but not VitK1, inhibited T-cell proliferation (Myneni and Mezey, 2018) and VitK2 derivatives inhibit proliferative responses and cytokine production by T-cells isolated from human PBMCs (Checker et al., 2011; Hatanaka et al., 2014). However, in human macrophagic THP-1 cells, VitK1 suppressed IL-6 production (Ohsaki et al., 2006), and dietary supplementation with VitK1 suppressed LPS-induced inflammation in rats (Ohsaki et al., 2006). The underlying mechanism may be linked to suppression of extracellular signal-regulated kinases (ERK), c-Jun N-terminal kinases (JNK) and NFκB in lymphocytes (Checker et al., 2011). Interestingly, VitK derivatives were also observed to increase Treg cell-frequencies in activated PBMCs (Hatanaka et al., 2014). In RBL-2H3, a model for mast cells, pre-incubation with VitK3 slightly inhibited degranulation and Ca2+ influx (Kawamura et al., 2006). Additionally, VitK3 treatment dose-dependently decreased leukotriene-secretion without affecting ERK or p38 phosphorylation (Kawamura et al., 2010). However, VitK3 is converted to VitK2 in the intestine but currently not allowed as a supplement for humans.

Taken together, these potential anti-inflammatory effects may point to an interesting role for VitK in allergy prevention/treatment. This is consistent with the findings from the Framingham Offspring Study in which inverse associations between VitK status and inflammatory biomarkers were observed (Shea et al., 2008). However, further investigation are warranted as data from the Danish National Birth Cohort suggest that maternal VitK intake increases the risk of admitted asthma and current asthma in 7-year-old children (Maslova et al., 2014).

### Luteolin and Quercetin

Fat-soluble phytochemicals can be separated into flavonoids and non-flavonoids. Flavonoids are polyphenolic components consisting of a three hydroxyflavone backbone and a diversity of side chains, making up 2,000 different components which are categorized in subgroups: flavones, flavanones, flavonols, flavononols, isoflavones, flavanols (catechins), and anthocyanidins. The average total human intake of flavonoids is 100–650 mg per day (Pan et al., 2010). Luteolin and quercetin act as anti-oxidants, with luteolin being more potent than several types of polyphenols including quercetin and resveratrol (Hofer et al., 2014). Quercetin however is also far more effective than VitE and VitC in lowering lipid peroxidation of albumin bound linoleic acid and PUFA oxidation (Fremont et al., 1998; Dufour and Loonis, 2007). Additionally, quercetin accumulates in mitochondria and protects against lipid peroxidase-induced mitochondrial damage (Fiorani et al., 2010). Both luteolin and quercetin are known for their anti-inflammatory capacity, lowering LPS-induced activation of AKT, MAPK (p38) and NFκB signaling cascades and pro-inflammatory cytokine release, possibly *via* disruption of lipid raft formation (Kim and Jobin, 2005; Kaneko et al., 2008). In addition, a metabolic breakdown product of quercetin was identified as competitive inhibitor of LOX (Borbulevych et al., 2004). However, pharmacokinetic studies showed poor absorption and fast metabolism of bioactive flavonoids like quercetin due to metabolization by gut microbiota. Currently, drug delivery systems are being developed to enhance delivery and bioavailability (Caddeo et al., 2019).

#### Luteolin and Quercetin and DCs and T-Cells

Luteolin completely blocked LPS-induced TNF-α and IL-12 expression in BMDCs, and i.p. injections prevented NFκB activation of LPS-activated PBMCs and splenocytes *in-vivo* (Kim and Jobin, 2005; Kim et al., 2019). Similarly, quercetin lowered TNF-α and IL-6 release by LPS-activated BMDCs, which was causally related to induced expression of secretory leukoprotease inhibitor (SLPI) known to suppress LPS-induced NFκB activation (Cavalcanti et al., 2014; De Santis et al., 2016). Quercetin improved extracellular iron transport contributing to an anti-inflammatory DC phenotype (Galleggiante et al., 2017). However, luteolin not only lowered IL-4 and IL-13 release by human basophils, but also IL-4 by human PBMCs with an IC50 at much lower dosing than quercetin (Hirano et al., 2004). Luteolin also inhibited murine and human inflammatory T-cell responses, while quercetin was ineffective (Verbeek et al., 2004). Furthermore, luteolin reduced effector DC maturation and T-cell responses *via* AKT/mTOR inhibition, while enhancing Treg responses in mice equally effective as rapamycin (Ye et al., 2019). However, in mice orally provided with lipid droplets containing quercetin and piperin, DC trafficking and consequent antigen-specific T-cell proliferation in lymph nodes was also strongly reduced (Delvecchio et al., 2015). A diet containing 0.1% quercetin prevented rhinovirus-induced exacerbation of COPD and airway inflammation (Farazuddin et al., 2018). In BMDCs quercetin enhanced disabled adapter protein (DAB) expression, linked to suppression of DC maturation, and reduced activation of NFκB and AKT signaling proteins (Lin et al., 2017). In addition, quercetin enhanced DAB2 expression in human DCs, and suppressed DC maturation *via* binding of AhR while inducing a regulatory DC phenotype directing regulatory T-cell development and TGF-ß release and inhibiting activation of Th-cells (Michalski et al., 2020). Hence, these studies clearly show the anti-inflammatory actions of both luteolin as well as quercetin by silencing DC activation and consequent T-cell proliferation, while supporting immunoregulatory functions (**Figure 3**).

#### Luteolin, Quercetin, and Mast Cells

Luteolin metabolites are characterized as IL-6 and COX-2 inhibitors (Quan et al., 2013) and luteolin inhibited secretion of pro-inflammatory cytokines like IL-1ß and TNF-α in HMC-1 cells (Jeon et al., 2014). These inhibitory effects may originate from reduced phosphorylation of MAPK complex, including JNK 1/2 and ERK 1/2 but not p38 MAPK. In HMC-1 cells, inhibition of the MAPK complex resulted downstream in reduced intracellular Ca2+ content, suppressed cytokine expression (IL-8, IL-6, TNF-α) and inhibition of NFκB (Kang et al., 2010). Inhibition of NFκB was also described for human CBMCs, resulting in reduced CCL2 release (Weng et al., 2015). Interestingly, luteolin can inhibit mast cell degranulation as well as its structurally related polyphenol quercetin at lower concentrations, indicating, like observed for the suppression of T-cell activation, a greater potency compared to quercetin (Baolin et al., 2004). Moreover, luteolin and its metabolite methlut were respectively 2,5 and 3 times more potent than the mast cell stabilizer chromolyn in human LAD-2 cells (Weng et al., 2015). Quercetin lowered mast cell degranulation of BMMCs and human cultured mast cells, while no effect was observed for the glycosylated form quercitrin (Kimata et al., 2000; Cruz et al., 2012). Protection by quercetin involves the inhibition of Lyn phosphorylation as assessed in human LAD2 cells and human basophilic KU812 cells. Reduced Lyn phosphorylation results indirectly in reduced phosphoinositide phospholipase C γ (PLCγ)-IP3R–Ca2+ signaling, ERK1/2 phosphorylation, IκB kinase (IKK) phosphorylation and NFκB expression (Kimata et al., 2000; Li et al., 2016; Ding et al., 2020) (**Figure 5**).

#### Luteolin and Quercetin in Pre-Clinical Allergy Models

In a guinea pig asthma model, luteolin and apigenin were more effective in suppressing acute and/or late phase allergen-induced airway hyperresponsiveness and inflammation than structurally related flavones and as effective as dexamethason (Lee et al., 2014). In a murine model for OVA-induced allergic asthma, dietary intervention with quercetin (10 mg/kg) for 5 days after sensitization but prior to and during challenge, reduced airway and systemic eosinophilia for more than 50% but less effective than dexamethasone (Rogerio et al., 2007). Quercetin (i.p.) supplementation for 3 days prior to OVA challenge reduced airway eosinophilia and AHR for more than 50%, while effectively lowering eosinophil peroxidase (EPO) activity and reducing pulmonary Th2/ILC2 markers GATA-3, IL-4 and IL-5, and enhancing Th1 marker T-bet (Park et al., 2009). In guinea pigs, both orally and pulmonary supplied quercetin reduced both immediate and late phase airway resistance, and inflammatory cell infiltration and histamine levels similar to effects of dexamethason (Moon et al., 2008). To improve bioavailability, a quercetin containing oil-in-water microemulsion was prepared (Rogerio et al., 2010). This was equally effective as dexamethasone in reducing airway eosinophilia in mice with OVA-induced asthma, while quercetin suspended in 0.5% carboxymethylcellulose was ineffective. The quercetin loaded micro-emulsion also suppressed Th2 cytokines IL-4 and IL-5 in BALF and NFκB activation in pulmonary tissue comparable to dexamethasone (Rogerio et al., 2010). Luteolin and quercetin have a great effect on attenuating allergic asthma symptoms and their bioavailability can be increased by encapsulation, making these bioactive micronutrients interesting candidates for allergy modulation.

### Resveratrol

Resveratrol (3,4,5-trihydroxy-trans-stilbene) is known for its strong anti-inflammatory potency. It is a ligand for the AhR receptor, which is known to favor the development of Tregs at the expense of inflammation-related Th17 effector cells development (Wang et al., 2013). Additionally, it can act as a Sirtin 1 agonist, enabling the deacetylation of transcription factors and lowering pro-inflammatory T-cell responses, amongst others NFκB-mediated inflammation (Malaguarnera, 2019; Delmas et al., 2020).

#### Resveratrol and Dendritic Cells and T-Cells

Resveratrol loaded nanostructured lipid carriers or free resveratrol suppressed NFκB activation in TNF-α activated moDCs (Barbosa et al., 2016). This effect was confirmed in another study showing impaired nuclear NFκB translocation, lowered co-stimulatory molecule expression and reduced DC induced allogenic T-cell proliferation (Silva et al., 2014). Similar results were obtained upon moDCs maturation with glycated albumin as a model for unwanted DC activation by advanced glycemic end products (Buttari et al., 2013). Resveratrol blocked DC activation and consequently T-cell proliferation and cytokine release, while largely preventing NFκB and MAPK, p38 and ERK activation (Buttari et al., 2013). Resveratrol also reduced CD80 and MHCII expression upon LPS-induced maturation of BMDCs, while increasing phagocytotic capacity and lowering DC-induced allogenic T-cell proliferation (Kim et al., 2004a). Monocytes exposed to resveratrol during differentiation to moDCs resulted in development of tolerogenic DCs instructing IL-10 secretion by allogenic T-cells (Svajger et al., 2010). Both splenic murine T-cell and B-cell proliferation and inflammatory cytokine release were suppressed by resveratrol, which coincided with enhanced regulatory IL-10 secretion (Sharma et al., 2007). Also, in T-cell receptor activated CD4+ T-cells, resveratrol blocked proliferation and reduced IL-2 receptor CD25 expression. Furthermore, IFN-γ release was blocked *via* suppression of the AKT/mTOR and MAPK(ERK) pathway, and T-cell metabolism was suppressed by modifying regulator 2 deacetylase (Sirt2) and p53. However, lower concentrations of resveratrol reduced proliferation but enhanced metabolic activity and induced IFN-γ release (Craveiro et al., 2017). The inhibition of T-cell proliferation and cytokine secretion by resveratrol was associated with increased Sirt1 expression (T-cell tolerance maintenance factor) upon resveratrol exposure (Zou et al., 2013). A recent study showed the interference of resveratrol with the interaction between PDL-1 and PD-1, known to contribute to Treg generation (Verdura et al., 2020). In a murine model of high fat (45%) induced regulatory T-cell dysfunction, resveratrol (0.06%) was indeed capable of restoring Treg levels and Treg related transcription factors, however this was achieved *via* prevention of ROS generation and stabilization of mitochondria preventing Treg cell death (Wang et al., 2014).

#### Resveratrol and Mast Cells

The strong inhibitory effect on mast cell degranulation by resveratrol was shown in BMMCs *ex-vivo*. The release of LTE4 and PGD2 was already inhibited upon administrating 10 µM resveratrol while histamine release was decreased at a dose of 100 µM. Interestingly, luteolin had to be administered tenfold less to achieve comparable effects (Baolin et al., 2004). Moreover, inhibition of IgE-mediated histamine release by orally administered resveratrol prior challenge was shown in BALB/c mice sensitized with anti-dinitrophenyl (DNP)-IgE (Han et al., 2013). Inhibition of mediator release by resveratrol is potentially induced by activation of negative signalling pathways, including Sirt1, which deacetylates several transcription factors. Activation of Sirt1 in BMMCs sensitized with DNP-IgE resulted in reduced phosphorylation of all MAPK, PLCγ1 and IKK. The effect of resveratrol was mostly inverted in Sirt1 knockout mice. (Li et al., 2017). Reduced phosphorylation of MAPK and PLCγ was also observed in human-derived skin mast cells, RBL-2H3 and HMC-1 (Koo et al., 2006; Kang et al., 2009; Han et al., 2015; Shirley et al., 2016). Overall, resveratrol can reduce mast cell degranulation by interfering with all three main pathways involved in cytokine/chemokine production, regulation of Ca2+ influx and mediator generation and release (Shirley et al., 2016).

#### Resveratrol in Pre-Clinical Allergy Models and Human Intervention Studies

Resveratrol not only abrogated IgE-allergen-induced ß-hexosaminidase and histamine release in RBL-2H3 and BMMCs, but also prevented OVA-induced diarrhea, temperature drop and anaphylactic symptom scores in OVA sensitized mice (Zhang et al., 2019). OVA-specific IgE levels were low, although IgG1 and IgG2a levels remained high. Serum histamine and murine mucosal mast cell protease 1 (mMCP-1) levels were significantly reduced, implying the potential of resveratrol to also lower mast cell degranulation *in vivo* in association with protection against food allergy symptoms (Zhang et al., 2019). These allergy protective effects were also shown in an OVA-induced food allergic model adding 0.01% resveratrol to the diet of mice (Okada et al., 2012). Resveratrol largely prevented sensitization since OVA-specific IgE was lowered for more than 50%, the challenge-induced drop in body temperature and the generation of allergen specific T-cell responses in both MLN and spleen was prevented (Okada et al., 2012). The resveratrol intervention inhibited DC-activation by lowering intracellular cAMP and expression of CD80 and CD86 on BMDCs (Okada et al., 2012). In a murine model of allergic asthma, resveratrol (daily *via* oral gavage, starting after the first sensitization) prevented OVA-induced pulmonary and systemic inflammation and type 2 responses (IL-5 and IL-13), while enhancing CD25+Foxp3+ Treg frequency and IL-10 miRNA expression in the lungs and downregulating miR34a expression (Alharris et al., 2018). Treatment of asthma *via* daily oral gavage with resveratrol reduced AHR and eosinophilia for 50% in association with lower type 2 cytokine levels in the BALF and reduced goblet cells and smooth muscle hyperplasia and collagen deposition as markers for tissue remodeling in the lung (Lee et al., 2017). Resveratrol inhibited pulmonary TGF-ß levels and mothers against decapentaplegic homolog 2 (SMAD2) activation, hence inhibiting a pathway known to contribute to airway remodeling (Lee et al., 2017). Although no randomized placebo controlled double blinded intervention studies in human have been done for the prevention of food allergy and asthma, resveratrol was tested in patients diagnosed with allergic rhinitis (n=151). Effects of a nose spray containing 0.1% resveratrol (0.6 µg/nostril/day) were compared with budesonide (400 µg/spray) or placebo for 1 month (Lv et al., 2018). After 2 and 4 weeks of treatment the nasal symptom score was reduced equally effective as the standard corticosteroid budesonide treatment. Hence, this study indicates the efficacy of resveratrol in suppressing allergic symptoms upon topical application in allergic rhinitis patients.

### Lycopene

Beyond flavonoids, other groups of fat-soluble phytochemicals like non-pro-vitamin A carotenoid lycopene and pro-vitamin A carotenoid ß-carotene also have immunomodulatory abilities, being the most abundant carotenoids in human nutrition (Rinaldi de Alvarenga et al., 2019). Among other carotenoids, ß-carotene can be converted into retinal, retinol (vitamin A) and bioactive all trans retinoic acid *via* beta-carotene oxygenase 1 or RALDH. Conversion of lycopene, the red pigment of tomatoes, is mainly done *via* ß-carotene oxygenase 2; it is less clear if it also effectively can be converted into VitA. Carotenoids, including lycopene are known ligands for RAR, RXR, and PPAR and may modify NFκB-induced inflammatory pathways *via* this way (Ruhl, 2013). The exact contribution of pro-vitamin A and non-pro-vitamin A carotenoids in relation to allergy risk remains to be revealed. Although the total amount of ß-carotene and lycopene were comparable between healthy volunteers and AD patients, levels of retinol (VitA) and all trans retinoic acid as well as lycopene metabolites lutein and zeaxanthin, were significantly reduced in plasma of AD patients (Lucas et al., 2018). Asthma patients with AHR had reduced ß-carotene and tocopherol blood levels (Wood and Gibson, 2010), while in stable asthma patients serum lycopene concentrations were reduced (Riccioni et al., 2006). The latter studies indicate the relevance of not only studying ß-carotene or VitA, but also lycopene in the context of allergic diseases. Lycopene is known as an anti-oxidant, but also has anti-inflammatory capacities. It can suppress pro-inflammatory signaling cascades like the MAPK and NFκB and reduce inflammation in humans (Kim et al., 2004b; Colman-Martinez et al., 2017).

#### Lycopene and DCs and T-Cells

In BMDCs, lycopene lowered LPS-induced maturation resulting in reduced CD80, CD86 and MHCII expression, increased phagocytotic capacity, and reduced DC-induced allogenic T-cell proliferation and cytokine release (Kim et al., 2004b). These results were obtained not only upon *in-vitro* exposure of BMDCs to lycopene, but also in splenic DCs obtained after *in-vivo* lycopene exposure and LPS challenge. Also, mitogen-induced activation of PBMCs isolated from healthy adults was dampened, and lycopene enriched liposomes lowered lymphocyte activation, proliferation, and cytokine release (Mills et al., 2012).

#### Lycopene and Mast Cells

The effect of lycopene on mast cell degranulation was studied by administrating lycopene, amongst other carotenoids, to RBL-2H3 cells prior stimulation. Overall, RBL-2H3 cells showed a 50% reduced ß-hexosaminidase release upon 4 h pre-incubation with 10 µM lycopene. Reduction by other carotenoids ranged from 75% (Fucoxanthin) to less than 10% (Lutein), indicating a moderate ability of lycopene to reduce degranulation in RBL-2H3 cells (Manabe et al., 2014). Feeding HR-1 hairless mice with lycopene prevented low mineral diet AD development in association with reduced number of skin mast cells (Hiragun et al., 2016). Overall, lycopene seems to have similar effects on mast cells like VitA reducing mast cell numbers by inhibition of mast cell maturation *via* binding to the RAR.

#### Lycopene in Pre-Clinical Allergy Models and Human Intervention Studies

Pre-clinical studies investigating ß-carotenoids or lycopene are mainly focused on AD and asthma development. In HR-1 hairless mice, ß-carotenoid and lycopene both reduced skin infiltration of inflammatory cells, partially restored the moisture level in the skin, but only lycopene lowered trans-epidermal water loss and mast cell counts (Hiragun et al., 2016). This indicates a protective role for carotenoids in the development of skin inflammation and barrier defects. In a murine model for OVA-induced asthma, i.p. lycopene injections of sensitized mice for 3 days before OVA challenge lowered AHR and eosinophilia for 50%, while normalizing IL-4 levels in the BALF and increasing IFN-γ, indicating a shift in T-cell response (Lee et al., 2008). Dietary supplementation for 14 days prior to and during OVA sensitization and challenge with lycopene (Lyc-O-Mato) partially prevented airway eosinophilia in mice, while OVA-specific IgG1 levels as marker for sensitization were not affected (Hazlewood et al., 2011). However, prophylactic lycopene treatment was unable to ameliorate AHR responses (Hazlewood et al., 2011). In humans, dietary supplementation of asthmatic adults with high vegetable/fruit intake (>7 servings of vegetables/fruits/day) slightly improved lung function, significantly reduced sputum eosinophil counts and increased the time to exacerbation in association with increased serum carotenoids, however, lycopene supplementation alone was not protective (Wood et al., 2012). Daily supplementation of 30 mg/mL lycopene (Lyc-o-Mato capsules for one week), however, was capable of protecting against an exercise-induced drop in airway function (forced expiratory volume in 1 s, FEV1) for more than 10% in almost half of the patients (n=21), while in the non-supplemented group only one fifth had a lower than 15% drop in FEV1. Supplementation resulted in a doubling of the serum lycopene levels (Neuman et al., 2000). The authors suggest lycopene to act as an anti-oxidant scavenging ROS that is released upon exercise and can provoke AHR in allergic asthma. Another intervention study showed reduced inflammation markers and enhanced total antioxidant capacity when 250 mg n-3 LCPUFAs were added to a lycopene rich tomato juice intervention (Garcia-Alonso et al., 2012).

#### Combining n-3 LCPUFAs With Bioactive Micronutrients in Allergic Disease

Beyond n-3 LCPUFAs, fat-soluble vitamins, polyphenols and carotenoids have been studied for their allergy protective effects *in-vitro* and in pre-clinical models. The interaction between n-3 LCPUFAs and bio-active fat-soluble nutrients have been investigated in a few studies, showing their combinatorial value. For example when combined with n-3 LCPUFAs, resveratrol enhanced the anti-inflammatory action of DHA, and synergized with EPA in reducing LPS activation of macrophages. Furthermore, EPA plus resveratrol downregulated the expression of multiple inflammatory and oxidative stress related genes, all indicating the combination of several components can make the difference (Pallares et al., 2012; Kuhn et al., 2018). These studies are promising but were not topic of clinical trials. In this regard only few studies have addressed a combination approach. In an open label study, a combination of n-3 and n-6 PUFA, VitE, VitC, zinc as well as multivitamin capsules for 4 months was found to reduce AD symptoms of adult severe AD patients with 50% (Eriksen and Kåre, 2006). However, a study using i.v. infusion with n-3 and n-6 PUFA containing lipid emulsions obtained a similar result, so the added value of combining the PUFA with the micronutrients remains to be revealed (Mayser et al., 2002). Combining VitD with a high dose of EPA/DHA (6 g daily) for 3 weeks did not improve lung function in exercise-induced asthma, while in a previous study intervention with only n-3 LCPUFAs was effective (Mickleborough et al., 2003; Price et al., 2015). Hence, in this patient group, the added value of VitD was not shown, and another strategy may be considered. Combining these type of bioactive nutrients for optimized efficacy in allergy prevention or attenuation of allergic symptoms is not often studied and requires additional studies.

## Discussion/Conclusion

Hence dietary intervention studies, aiming to determine effects of specific components on allergy prevention and/or amelioration of symptoms, mainly focus on a single active ingredient or food group per trial. n-3 LCPUFAs for example may be able to reduce the risk of atopic disease, albeit only in high doses starting already during early pregnancy (Dunstan et al., 2003a; Furuhjelm et al., 2009; Best et al., 2018). This approach may also help to prevent childhood asthma development and is especially indicated for those having low serum n-3 LCPUFAs levels due to low dietary intake or a specific *FADS* genotype (Tanaka et al., 2009). However, many other dietary components like fat-soluble nutrients possess anti-inflammatory activity targeting multiple cellular targets, potentially acting in an additive or synergistical manner to n-3 LCPUFAs which may help to optimize their full potential and gain efficacy in allergy prevention or treatment (**Figure 2**).

Despite their acknowledged anti-oxidant and anti-inflammatory capacities, therapeutic use of natural/biological polyphenols is thought to be limited due to their low bioavailability. Although the food matrix and oil composition can provide improved bioavailability, pharmaceutical concepts are under development to enforce their therapeutic value. Nanoparticle developments for enhanced gastrointestinal delivery and uptake or skin application is ongoing and promising, including diverse types of lipid-rich nanoparticles like liposomes or nanoparticles with additional functional groups as the recently developed quercetin loaded fucoidan/chitosan nanoparticles (Barbosa et al., 2019). Solid lipid nanoparticles containing n-3 LCPUFAs and esterified resveratrol are also being generated for pharmaceutical purposes (Serini et al., 2018). This allows combined delivery and solid lipid nanoparticles containing DHA and resveratrol indeed effectively abrogated the chemically induced inflammatory response in keratinocytes (Serini et al., 2019). Furthermore, unique nanoparticles (e.g. specific liposomes) loaded with antigen and quercetin achieved antigen specific immune silencing (NFκB inhibition similar to chemical inhibitor Bay) (Capini et al., 2009), showing the immunomodulatory and anti-inflammatory potency of these bioactive micronutrients in an antigen-specific manner. This is also exemplified by topical application of quercetin or resveratrol in murine models, being able to block skin inflammation in a contact hypersensitivity model (Carbone et al., 2018).

Overall, Mediterranean diet derived components like n-3 LCPUFAs, fat-soluble vitamins, polyphenols and carotenoids including lycopene are known for their anti-inflammatory and/or anti-allergic capacities with diverse underlying mechanisms, indicating their potencies to improve allergy protection by combining these elements. This hypothesis needs further investigation to reveal their capacity in fighting the progression of NCDs and especially the rising incidence of allergic disorders in industrialized countries.

## Author Contributions

AH, AE, and LW have written the review. JG has critically read the review. All authors contributed to the article and approved the submitted version.

## Conflict of Interest

AE is employed at the University Medical Centre Utrecht. JG is head of the Division of Pharmacology, Utrecht Institute for Pharmaceutical Sciences, Faculty of Science at Utrecht University and partly employed by Danone Nutricia Research B.V. AH and LW are employed at the Division of Pharmacology of the Utrecht University that collaborates within a strategic alliance with Danone Nutricia Research B.V.

## Glossary

**Table:**

25(OH)D3	25-dihydroxyvitamin D3
aOR AA	Adjusted odds ratio arachidonic acid
AD	atopic dermatitis
AhR	arylhydrocarbon receptor
AHR	airway hyperreactivity
ALA	alpha-linolenic acid
AMPK	5’ adenosine monophosphate activated protein kinase
BALF	broncho alveolar fluid
Bcl-6	B-cell lymphoma 6
BMDCs	bone marrow derived DCs
BMMCs	bone marrow-derived mast cells
cAMP	3’-5’-cyclic adenosine monophosphate
CBMCs	cord blood derived mast cells
CBMNCs	cord blood mononuclear cells
CCL5	C-C motif chemokine ligand 5
CCR	C-C chemokine receptor
CXCR	C-X-C chemokine receptor
CMA	cow’s milk allergy
COX	cyclo-oxygenase
DCs	dendritic cells
DGLA	di-homo-gamma-linolenic acid
DHA	docosahexaenoic acid
DNP	anti-dinitrophenyl
DSS	dextran sulphate sodium
EPA	eicosapentaenoic acid
EPO	eosinophil peroxidase
ERK	extracellular signal-regulated kinase
ϵGLT	epsilon germline transcript
*FADS*	fatty acid desaturase
FEV1	forced expiration volume in 1 second
Foxp3	forkhead box p3
GPR120	G-protein coupled receptor 120
HDM	house dust mite
IκB	inhibitor of kappa B
IFN	Interferon
IKK	IkB kinase
IL	Interleukin
ILC2s	innate lymphoid cells 2
JNK	janus kinase
LA	linoleic acid
LCPUFAs	long chain poly-unsaturated fatty acids
LOX	Lipoxygenase
LPS	Lipopolysaccharide
LTB4	leukotrien B4
MAPK	mitogen activated protein kinase
MHCII	major histocompatibility complex II
MLN	mesenteric lymph nodes
mMCP-1	murine mucosal mast cell protease 1
moDCs	monocyte derived DCs
mRNA	messenger RNA
mTOR	mammalian target of rapamycin kinase
NCD	non-communicable diseases
NFkB	nuclear factor kappa B
NOD2	nucleotide binding oligomerization containing protein 2
Nrf2	nuclear factor erythroid-derived 2
OVA	ovalbumin
PAF	platelet-activating factor
PBMCs	peripheral blood mononuclear cells
PD1	Programmed cell death 1
PGE2/PGD2	Prostaglandin E2/D2
PKB/C	protein kinase B/C
PLA2	phospholipase A2
PLCγ-IP3R	Phospholipase C gamma- inositol 1,4,5-triphosphate receptor
PPAR	peroxisome proliferator-activated receptor
RA	retinoic acid
RALDH	retinal dehydrogenases
RAR	retinoic acid receptor
RARE	RAR response elements
RXR	retinoid x receptor
RXRE	RXR response elements
RvE1	resolvin E1
ROS	reactive oxygen species
SCORAD	scoring atopic dermatitis
Sirt2	regulator 2 deacetylase
SMAD2	mothers against decapentaplegic homolog 2
Srcin 1	Src-kinase-signaling inhibitor 1
STAT6	signal transducer and activator of transcription 6
Syk	spleen tyrosine kinase
TGFβ	transforming growth factor beta
Th	T helper cells
TLR	toll-like receptor
Treg	regulatory T cell
TEWL	trans-epidermal water loss
TSLP VDR	thymic stromal lymphopoietin vitamin D receptor
VDRE	vitamin D responsive element
VitA	vitamin A
VitD	vitamin D
VitE	vitamin E
VitK	vitamin K
